# Health motivations and perceived barriers are determinants of self-care behaviour for the prevention of hypertension in a Malaysian community

**DOI:** 10.1371/journal.pone.0278761

**Published:** 2022-12-07

**Authors:** Paulina Pei Suu Tan, Ryand Singh Sandhu, Shamsul Mohd Zain, Deborah Hall, Ngiap Chuan Tan, Hooi Min Lim, Faiz Daud, Yuh-Fen Pung

**Affiliations:** 1 School of Pharmacy, University of Nottingham Malaysia, Semenyih, Selangor, Malaysia; 2 Department of Pharmacology, University Malaya, Kuala Lumpur, Malaysia; 3 Department of Psychology, Heriot-Watt University Malaysia, Putrajaya, Malaysia; 4 Department of Research, SingHealth Polyclinics, Singapore, Singapore; 5 Department of Primary Care Medicine, University Malaya Medical Centre, University of Malaya, Kuala Lumpur, Malaysia; 6 Department of Community Health, University Kebangsaan Malaysia Medical Centre, Kuala Lumpur, Malaysia; Keele University, UNITED KINGDOM

## Abstract

**Introduction:**

Self-care behaviour is fundamental in preventing hypertension in the general population. According to the Health Belief Model, health beliefs and perceptions influence the success in adopting disease prevention strategies. While factors influencing hypertension self-care behaviour have been examined previously in patient populations, they have not been assessed in the general community.

**Methods:**

This was a cross-sectional study conducted between 12 June 2020 to 26 July 2021. An online survey was administered via email and social media to Malaysians in the Selangor and Kuala Lumpur communities. Respondents were over 18 years old, without a formal diagnosis of hypertension. The survey evaluated hypertension knowledge, Health Belief Model constructs, self-care behaviour frequency, and motivators and barriers to self-care behaviour. Multiple linear regression was performed to determine the main predictors of self-care behaviour, and descriptive statistics were used to characterise motivators and barriers of each self-care behaviour.

**Results:**

Only health motivations (β = 0.217, *p* < 0.001) and perceived barriers (β = 0.571, *p* < 0.001) significantly influenced self-care behaviour. Maintaining a healthy diet, regular physical activity and blood pressure checks need to be improved in the community, particularly in reducing salt and calorie intake. Lack of time, limited choices and laziness are the biggest challenges that need to be tackled in adopting a healthy diet and an active lifestyle in the community. Many are ignorant towards their health status, therefore, do not prioritize blood pressure screenings, suggesting a need to enhance community blood pressure checks for early diagnosis of hypertension.

**Conclusion and implications:**

Motivations and barriers were the main determinants of self-care behaviour in the Selangor and Kuala Lumpur community. Targeting these aspects of self-care behaviour should be considered when developing interventions and education programmes tailored to local cultural, environmental and personal factors, to more effectively reduce the hypertension prevalence and burden.

## Introduction

Cardiovascular disease (CVD) is the leading cause of death worldwide, with most deaths attributed to coronary heart disease or stroke [1]. Hypertension is a large risk factor for CVDs with two-thirds living in low- and middle-income countries, and contributes to overall premature death [1]. Malaysia is no exception. Despite the capacity to control and prevent hypertension through lifestyle modifications or hypertension self-care behaviours, CVDs such as ischaemic heart disease was the biggest causes of death Malaysia in 2020 [2]. The National Health and Morbidity Survey (NHMS) 2019 of a representative sample of 14,965 Malaysian adults reported an overall hypertension prevalence of 30% (15.9% known hypertension, 14.1% raised blood pressure among unknown hypertension) [3]. More recently, the Malaysian Community Salt Study (MyCoSS) reported a 49.39% overall prevalence of hypertension (95% CI 44.27–54.51) in a representative sample of 1047 adults [4]. A secondary analysis of the NHMS 2015 of a representative sample of 15,738 Malaysian adults reported a staggering 66.8% overall prevalence of increased blood pressure (95% CI: 65.6–68.0), comprising 45.8% prehypertension (95% CI: 44.66–46.97), 15.1% Stage 1 hypertension (95% CI: 14.34–15.92) and 5.9% Stage 2 hypertension (95% CI: 5.44–6.41) [5]. Mahadir Naidu and colleagues described this as an epidemic [5]. The collective survey findings show that a large proportion of the adult population have elevated blood pressure and are at risk of developing CVDs if their condition remains uncontrolled.

Here, hypertension self-care behaviours is defined as behaviours which help to control blood pressure and deter the onset of hypertension. These behaviours include various lifestyle modifications such as daily intake of less than 5 g salt and at least five servings of fruits and vegetables, reducing fat intake, regular physical activity, limiting alcohol consumption, practicing non-smoking, and regular monitoring of blood pressure [6–8]. Regular practice of hypertension self-care behaviours prevents development of hypertension, helps improve blood pressure management and can aid early detection of hypertension to prevent long-term complications. Hence, emphasis on prevention through improving hypertension self-care behaviours should take precedence to reduce the overall prevalence of hypertension and alleviate its implicated socioeconomic burdens [9–12].

Evidence-based development of interventions framed within a validated theoretical model are preferable to those without since they are often more clinically relevant and cost effective [13]. Despite the effectiveness of theoretical model-based interventions, the literature on self-care practices to prevent hypertension and manage blood pressure in Malaysia is rather sparse. Four studies were identified which were all exploratory and descriptive rather than hypothesis driven, and these studies failed to frame their research questions in the context of any health behavioural model or theory [14–17]. Poor blood pressure control evidenced by the high prevalence suggest opportunities to improve management and prevention interventions in Malaysia. Hence, reducing the knowledge gap in the application of theoretical-models in hypertension prevention interventions is worthwhile [18].

**Fig 1:**
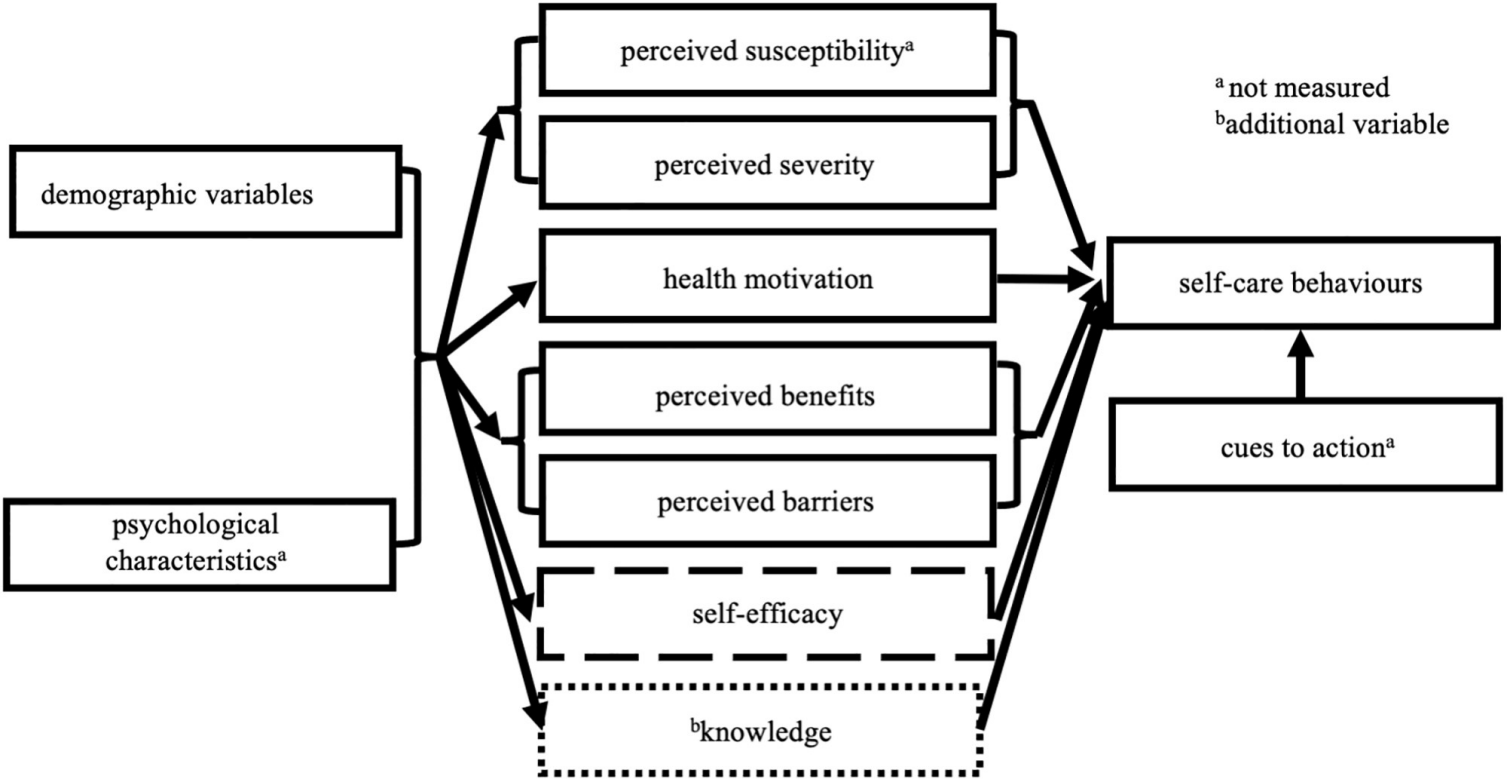
A version of the Health Belief Model (HBM) in adopting hypertension self-care behaviours in the present study. Self-efficacy was added to the model in the mid-1980s and so is illustrated by a dashed line. Knowledge was hypothesized to be an additional variable in influencing self-care behaviour due to its association with blood pressure control in our study.

With respect to understanding the success or failure of disease prevention strategies and screening, the Health Belief Model (HBM) is widely applied today, despite its conception in the 1950’s [19–21]. The HBM derives from psychological and behavioural theory and proposes that an individual’s course of action often depends on their perceptions of the benefits and barriers related to a certain health behaviour. The version of the HBM adopted here posits that the likelihood a person will adopt a positive health behaviour is determined by six factors: i) perceived susceptibility (the risk of becoming hypertensive), ii) perceived severity (how serious hypertension is seen to be), iii) health motivation (concern about health), iv) perceived benefits (the effectiveness of preventative actions), v) perceived barriers (obstacles to performing the positive health behaviour), and vi) self-efficacy (confidence in ability to successfully perform the desired behaviour) (Fig 1, refer to methods). These six factors can be modified by socio-demographic variables (such as age, sex, level of education) or psychological characteristics (such as personality), while cue to action can modify health behaviours. In the present study, self-efficacy and hypertension knowledge were included in the model based on its known association with the adoption of hypertension self-care behaviour [15, 17, 22–27]. Out of these six factors, perceived susceptibility was found to be the weakest predictor of behaviour and had close to no relationship with positive health behaviour [28]. Therefore, perceived susceptibility was not measured in the present study.

This cross-sectional study evaluated predictive strength of six factors (i.e. perceived severity, health motivation, perceived benefits, perceived barriers, self-efficacy and knowledge) in regards to hypertension self-care behaviour in a convenience sample of the Malaysian community.

## Methods

### Ethics approval

Ethical approval was approved by the Science and Engineering Research Ethics Committee (SEREC) at the University of Nottingham Malaysia (ref: PT110520). Electronic consent was obtained from all participants before starting the anonymous online survey. Participants who denied consent were redirected to the end of the survey. Additional information regarding the ethical, cultural, and scientific considerations specific to inclusivity in global research is included in the Supporting Information (S1 Checklist).

### Sample size, eligibility and recruitment

Participants were recruited from 12 June 2020 to 26 July 2021 through distribution of the online survey link, via email and social media. The inclusion criteria was Malaysian adults aged 18 years and above, no formal diagnosis of hypertension, and residing in Selangor or Kuala Lumpur. During provision of electronic consent, a series of yes-or-no questions evaluating the participants’ understanding of their participation in the study and their eligibility were asked. Those that were ineligible were automatically redirected to the end of the survey.

Sample size was estimated using G* Power version 3.1.9.4 adopting an *a priori* F test with the following assumptions: effect size = 0.15, margin of error = 5% and number of predictors = 5. Following this, the minimum sample size required to detect a power of 95% was estimated to be 138. Assuming a 10% rate of non-completion of the full survey, the target sample size was 151.

### Survey

The survey instrument was informed by the Health Belief Model (HBM) (Fig 1) and administered in an online survey using Qualtrics (Seattle, USA) in English, Bahasa Malaysia, and Mandarin (S1 Appendix). Items spanned of the six predictors of interest (i.e. perceived severity, health motivation, perceived benefits, perceived barriers, self-efficacy and knowledge about hypertension), the outcome (i.e. current self-care behaviours) [29], and participant socio-demographics [30, 31].

Perceived severity was measured by a single item (“How dangerous is hypertension to your health?”) with a 3-point ordinal scale response option (3 = extremely, 2 = somewhat, 1 = not at all). Perceived benefits was measured by two items (“Would lowering high blood pressure improve a person?”, “Can lowering blood pressure even a little bit improve health?”) with a 3-point ordinal scale response option on capability (3 = yes, 2 = somewhat, 1 = no). Self-efficacy was measured by three items (“Can people do things to control their blood pressure?” with a 3-point ordinal scale response option on capability (3 = yes, 2 = somewhat, 1 = no).

Health motivations, perceived barriers and current self-care behaviours were measured by the Hypertension Self-Care Profile (HTN-SCP) [32]. Each HTN-SCP scale includes 20 items written at 6^th^ grade reading level. Response options use a 4-point ordinal scale with increments of agreement or compliance with each statement (4 = “very important”/ “very easy”/ “always”, 3 = “fairly important”/ “fairly easy”/ “often”, 2 = “important”/ “fairly difficult”/ “sometimes”, 1 = “not important”/ “very difficult”/ “rarely/never”). Hence, higher scores indicate higher levels of self-care behaviour etc. The original 20-item tool was modified by omitting five statements that were irrelevant to a non-hypertensive group (such as “take your blood pressure medication”). This left a total of 15 statements about self-care behaviour concerning five broad categories: salt and calorie intake, fruit and vegetable intake, physical activity, alcohol intake and blood pressure screening. For each statement, participants were asked about their current health behaviour (how frequent?), its health motivation (how important?) and any perceived barrier (how difficult?). Participants were asked to select a maximum of three options from three to five pre-existing motivations (such as relieve stress, improve or maintain health, lose weight) or three to five barriers (such as no time, limited food choices, lazy) or they were able to write down an additional factor not on the list. The pre-existing options were based on responses in previous studies conducted in Malaysian populations [17, 33, 34]. Motivators and barriers for were not evaluated for smoking as a large majority (nearly 90%, S1 Table) of participants practiced non-smoking, and extensive research has been done in investigating factors that influenced smoking attitudes in the nationwide population [35–38]. For each subscale, the maximum score was 60 and minimum score was 15 on an interval scale.

Knowledge about hypertension was assessed using items taken from Oliveria et al (2005) [39] and translated into Malay by Mohammed et al (2019) [7]. This knowledge subscale comprised: what hypertension means, how dangerous it is to health, impact of lowering blood pressure (2 points), how to interpret blood pressure scores (5 points), and whether behaviour can make a difference [39]. The maximum score was 10 and minimum score was 0.

Socio-demographic assessments included age, sex, ethnicity, body mass index (BMI), level of education, and household income.

### Data analysis

Statistical analysis was carried out using SPSS version 27.0. The sample was described using descriptive statistics of perceived severity, health motivation, perceived barriers, self-efficacy and hypertension knowledge that influence self-care behaviour.

To determine which factors predicted self-care behaviour, multiple linear regression was performed. Kaiser-Meyer-Olkin (KMO) (> 0.5) and Bartlett’s test (p < 0.05) (S2B Table) was used to confirm homogeneity of variance. Variance Inflation Factors (VIF) (1.128–1.727), and tolerance values (0.579–0.886) (S2C Table) were used to demonstrate the absence of multicollinearity between variables. To analyse health motivation and perceived barriers of self-care behaviour, the frequency of the five behaviour groups in section 4 were stratified into ‘always/often’, and ‘rarely/sometimes’, based on the average of each HTN-SCP statement in the behaviour groups. Descriptive statistics of the selected barriers reported were filtered by those who ‘rarely’ or ‘sometimes’ carried out the respective behaviour; similarly, statistics of motivators were filtered by selection of ‘often’ or ‘always’. A *p* < 0.05 was considered as statistically significant in this study.

## Results

### Participants

There were 260 respondents, but 22 did not complete the survey, indicating a completion rate of 91.5%. After excluding these from the data set, this left a total of 238 for analysis. Participant demographics is summarised in Table 1. The mean age of the participants was 32.0 (SD 10.9) years old. There were more female participants (60.9%), with Chinese (51.3%) participants as the most prevalent ethnic group, followed by Malays (38.7%), Indians (8.8%), and other local minority groups (1.3%) such as Sikh, and other indigenous tribes. The participants predominantly achieved university level qualifications (85.3%), while the rest had received education up to pre-university levels (14.7%). A majority of participants were from the middle class (40.3%) with a monthly household income of RM 4000 to RM 9500, followed by the upper class with monthly income of more than RM 9500 (25.6%), lower class with monthly income between RM 1000 to RM 3999 (23.9%), and extreme low class with less than RM999 monthly income (10.1%). Most participants had normal weight (35%) or were overweight (31.5%); while a smaller proportion were obese class I (13.2%), underweight (11.5%), and obese class II (8.1%).

**Table 1:** Participant socio-demographics and self-care behaviour.

Variables	n (%)	Mean (SD)	Behaviour score Mean (SD)
**Age**		32.0 (10.9)	
**Sex** ^**a**^			
Male	93 (39.1)		38.5 (7.9)
Female	145 (60.9)		39.8 (8.3)
**Ethnicity** ^**b**^			
Malay	92 (38.7)		39.0 (8.1)
Chinese	122 (51.3)		39.3 (8.0)
Indian	21 (8.8)		40.4 (10.1)
Others	3 (1.3)		39 (5.6)
**BMI class** ^**b**^			
Underweight	27 (11.5)		40.7 (9.5)
Normal	84 (35.7)		38.6 (8.5)
Overweight	74 (31.5)		40.0 (8.3)
Obese class I	31 (13.2)		39.1 (1.4)
Obese class II	19 (8.1)		38.5 (5.7)
**Education level** ^**b**^			
Pre-university	35 (14.7)		37.7 (7.6)
University	203 (85.3)		39.6 (8.3)
**Household income (per month)** ^**b**^			
< RM 999	24 (10.1)		38.5 (7.8)
RM 1000 –RM 3999	57 (23.9)		37.5 (7.2)
RM 4000 –RM 9500	96 (40.3)		39.6 (8.5)
> RM 9500	61 (25.6)		40.8 (8.5)

### Health behaviour model predictors

Participants understood the severity of hypertension (mean = 2.6, SD = 0.5, out of 3), the benefits of controlled blood pressure (mean = 4.7, SD = 0.7, out of 6) and the ability to prevent hypertension, indicated by self-efficacy (mean = 6.6, SD = 0.8, out of 9), and had reasonably good hypertension knowledge (mean = 6.8, SD = 2.2, out of 10), (Table 2). Mean scores for health motivation and perceived barriers were modest at 46.0 (SD = 9.4) out of 60, and 45.3 (SD = 8.2) out of 60, respectively.

**Table 2:** Hypertension knowledge and Health Belief Model (HBM) predictor scores.

Scores	Minimum	Maximum	Mean	SD
Perceived severity	1	3	2.6	0.5
Health motivation	15	60	46.0	9.4
Perceived benefits	1	6	4.7	0.7
Perceived barrier	15	60	45.3	7.3
Self-efficacy	1	3	2.0	0.2
Hypertension knowledge	0	10	6.8	2.2

### Frequency of self-care behaviour

**Fig 2:**
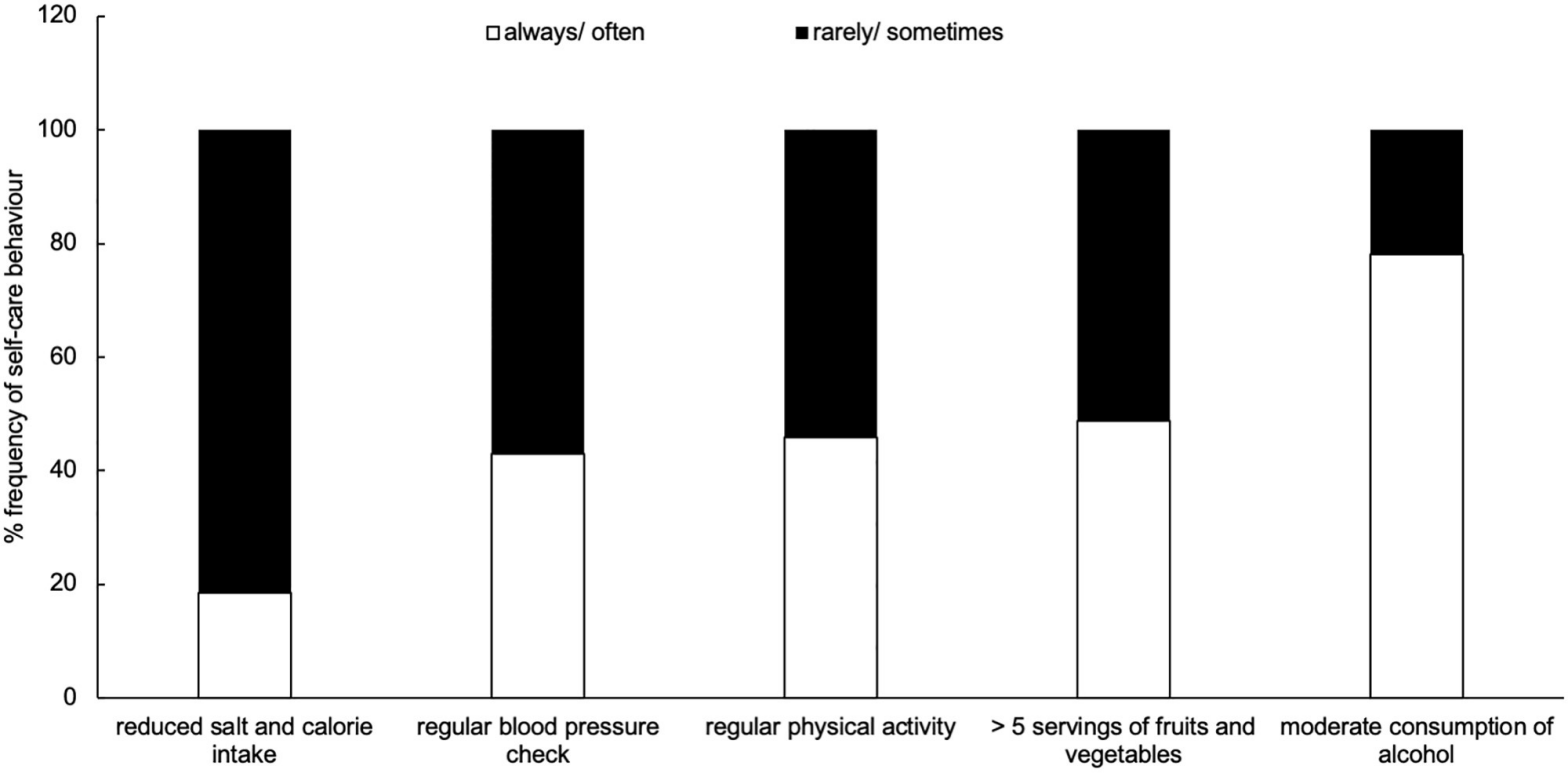
Percentage frequency of self-care behaviour among the community in Selangor and Kuala Lumpur (N = 200).

The mean score for self-care behaviour was 39.3 (SD = 8.2) out of 60. Categorizing self-care behaviours into five broad categories showed that practicing reduced salt and calorie intake (rarely/sometimes = 81.5%) was the most challenging in the Selangor and Kuala Lumpur communities (Fig 2). Consuming more than 5 servings of fruits and vegetables, regular physical activity, and frequency of regular blood pressure screening (checks at least once a year) was inadequate at 51.3%, 54.2%, and 57.1%, respectively for selection of ‘rarely’ or ‘sometimes’ (Fig 2). Moderate consumption of alcohol (always/often = 78.2%) was frequently carried out by the community (Fig 2).

### Health behaviour model predictors of self-care behaviour

The multiple linear regression model with the six predictor variables (i.e. perceived severity, health motivation, perceived benefits, perceived barriers, self-efficacy and knowledge about hypertension) indicated significant predictors of self-care behaviour (R^*2*^ = 0.487, F (6,231) = 38.541, *p* < 0.001). Thus, 48% of the variation in self-care behaviour was explained by the six measured predictors. Considering the individual contributions to self-care behaviour, only health motivations and perceived barriers significantly contributed to the outcome (Table 3).

**Table 3:** R socio-demographic factors, hypertension knowledge, health belief model constructs, and self-care behaviour.

Variables	β	95.0% CI		*t*	*p*-value
		lower	Upper		
Perceived severity	-0.017	-1.743	1.222	-0.346	0.729
Health motivation	0.217	0.099	0.278	4.142	< 0.001**
Perceived benefits	-0.067	-1.895	0.404	0.578	0.563
Perceived barriers	0.571	0.532	0.758	11.246	< 0.001**
Self-efficacy	0.030	-2.926	5.395	0.579	0.563
Hypertension knowledge	0.080	-0.148	0.278	1.315	0.190
**Adjusted R^*2*^ = 0.487**					

** *p* < 0.001

### Motivators for appropriate self-care behaviour

The strongest motivator to hypertension self-care behaviour was to maintain and improve health (range = 61.6%–95.5%), overarching all diet and physical activity-based self-care behaviours, excluding regular blood pressure measurements (Table 4). Self-care behaviours pertaining to diet such as reduced salt and calorie intake, and consuming more than five servings of fruits and vegetables daily had similar motivators, where disease prevention (range = 60%–68.8%) was the second highest motivator, followed by intention of weight loss (40%–47.3%), and personal preference (range = 1.8%–8.6%). For regular physical activity, another top motivator was the desire to keep fit (76.6%), followed by exercising as stress relief (70.1%), for good appearance (28%), and motivation from friends and family (8.4%). Half of the respondents (52.9%) avoided or practiced moderate alcohol consumption due to religious reasons, 37.2% disliked the taste of alcohol, and the remaining 7% had reasons such as alcohol allergy or high cost of alcohol. Motivators for regular blood pressure measurements were most dissimilar to the other diet or physical activity-based self-care behaviours. Routine or opportunistic screening such as blood pressure measurements during doctor’s appointments (68.4%) and to smaller extent free-community screenings (27.8%) encouraged regular blood pressure measurements. A quarter of respondents (25.3%) screened their blood pressure upon family’s request, while only a small group voted that owning a home blood pressure monitoring machine (3.8%) motivated regular blood pressure monitoring. Other reasons (11.4%) included blood donation, experiencing hypertension symptoms or for personal knowledge (Table 4).

**Table 4:** Motivators to hypertension self-care behaviours. Motivators were obtained from participants whom ‘always’ or ‘often’ carry out these behaviours.

Motivators	count	%
**reduced salt and calorie intake (always/ often = 141)**		
maintain health	26	74.3
prevent disease	21	60.0
lose weight	14	40.0
others	3	8.6
**> 5 servings fruits and vegetables (always/ often = 112)**		
maintain and improve health	107	95.5
prevent disease	77	68.8
lose weight	53	47.3
tastes good	11	9.8
others	2	1.8
**regular physical activity (always/ often = 107)**		
maintain and improve health	85	79.4
to keep fit	82	76.6
relieve stress	75	70.1
to keep slim for beauty purposes	30	28.0
motivation from friends and family	9	8.4
**moderate alcohol consumption (always/ often = 172)**		
know that alcohol is bad for health	106	61.6
religion	91	52.9
dislike the taste of alcohol	64	37.2
others	12	7.0
**regular blood pressure check (always/ often = 26)**		
part of routine check when visiting the doctor	54	68.4
free community checks	22	27.8
family request	20	25.3
others	9	11.4
owns BP machine	3	3.8

### Barriers to appropriate self-care behaviour

#### Barriers to diet-based self-care behaviours

Barriers to maintaining a healthy diet by controlling salt and calorie intake, or consuming adequate fruits and vegetables were similar. Common barriers included the lack of time prepare healthy foods due to busy schedule, the lack of healthy food options at work place or eating out, and to a lesser extent the cost of healthy foods (Table 5). For reduced salt and calorie intake, the lack of time to cook or prepare healthier meals was the largest barrier (48%), followed by limited choice (32.6%), cost (31%), influence by family diet (31%), and personal preference (27%). Interestingly, a large majority (79.1%) of respondents indicated that there were limited choices of meals with fruit and vegetables at their school or workplace, resulting in the inability to meet the recommended daily consumption. Others disliked the taste of fruits or vegetables (20.9%) and thought that fruits were expensive (6%). Remaining barriers for sufficient fruit and vegetable consumption (11.9%) included reasons such as influence by family diet, thought that five servings a day were too much, and some acknowledged that most local foods are traditionally high in carbohydrates, therefore, more effort was needed to ensure a healthy diet.

**Table 5:** Barriers against hypertension self-care behaviours. Barriers were obtained from participants whom ‘sometimes’ or ‘rarely/never’ carry out these behaviours.

Barriers	count	%
**Reduced salt and calorie intake (sometimes/ rarely/ never = 129)**		
no time	62	48.1
limited choice	42	32.6
expensive	40	31.0
family diet	40	31.0
prefer salty and fried foods	35	27.1
others	9	7.0
**> 5 servings fruits and vegetables (sometimes/ rarely/ never = 67)**		
limited choices	53	79.1
dislike taste	14	20.9
others	8	11.9
expensive	4	6.0
**regular physical activity (sometimes/ rarely/ never = 108)**		
no time/ busy work schedule	78	72.2
lazy	78	72.2
lack of safe space	27	25.0
others	5	4.6
**moderate alcohol consumption (sometimes/ rarely/ never = 22)**		
festive reasons	13	59.1
stress relief	8	36.4
others	7	31.8
peer pressure	2	9.1
**regular blood pressure check (sometimes/ rarely/ never = 90)**		
never thought about it	64	71.1
healthy and don’t see a need	49	54.4
health anxiety	14	15.6
others	9	10.0

#### Barriers to regular physical activity, moderate alcohol consumption, and regular blood pressure screening

The lack of time to exercise (72.2%) and laziness (72.2%) were the greatest barrier to regular physical exercise (Table 5). Lack of time and laziness were followed by the lack of safe space to exercise (25%) and other reasons such as walking difficulty or family responsibilities (Table 5). Barriers to moderate alcohol consumption were celebrations during festive seasons (59.1%), stress relief (36.4%), peer pressure (9.1%), and other reasons such as personal preference (Table 5). For regular blood pressure screening, a large majority never thought about it (71.1%), and thought that it was unnecessary as assumed that they were healthy (54.4%) (Table 5). Some were anxious about knowing their health status (15.6%), while other reasons included the additional expense and inconvenience for blood pressure measurements (10%) (Table 5).

## Discussion

To our knowledge, this study is one of the first of its kind to evaluate determinants of self-care behaviour for hypertension prevention in the general community based on the HBM. The present study showed that perceived barriers and health motivations were the strongest determinants of self-care behaviour in a convenience sample. We also observed that although participants knew that self-care behaviours could maintain health and prevent diseases, they were unable to practice these behaviours regularly due to various identified barriers.

### Predictors of hypertension self-care behaviours: Prevention versus treatment

Self-care behaviour for blood pressure management is similar for both treatment and prevention of hypertension in terms of lifestyle modifications, where only medication adherence is specific to hypertensive patients. The HBM was selected as the framework of this study as it is particularly well suited to health conditions that can be prevented or ameliorated through individual action and so hypertension is an excellent candidate [20, 40–42]. Numerous studies have confirmed associations between HBM predictors and self-care behaviours particularly for blood pressure control in hypertensive patients [20, 40, 41]. For this reason, we hypothesized that the HBM would be a suitable framework for this study to determine predictors of self-care behaviour in the general community.

Previous studies demonstrated that at least five HBM predictors were significantly associated with self-care behaviour in blood pressure management in hypertensive patients [20, 40, 41]. For example, a cross-sectional study confirmed that perceived susceptibility, perceived severity, perceived benefits, perceived barriers and self-efficacy accounted for 47.0% of the total variance in self-care behaviours in a convenience sample of young and middle-aged adults with hypertension recruited through two teaching hospitals in China [20]. Another study of treated hypertensive patients in China showed a similar profile where all five HBM factors explained 50.5% of the variance in adherence to antihypertensive medication, with an overall prediction accuracy of 86.2% [41]. Similarly, a cross-sectional study conducted in Iran reported that those hospital patients who perceived high susceptibility, severity and benefit had better adherence to antihypertensive medication compared those with low scores on all three factors [40]. These results differed from the regression model in this study where only health motivations and perceived barriers significantly associated with self-care behaviour. Based on a meta-analysis of 18 studies (2,702 subjects) where the outcome of half the studies were related to treatment adherence and the remaining for disease prevention, self-care behaviours that involved medication adherence were more positively related to perceived susceptibility and severity, while behaviours to prevent a negative health outcome were most associated with perceived benefits and barriers [28]. Hence, differences in predictors associated with self-care behaviour may be attributed to whether participants were practicing self-care behaviour for treatment or prevention of hypertension.

Our study demonstrated that knowledge about hypertension was not associated with self-care behaviour. This was inconsistent with previous studies reporting a positive influence of knowledge about hypertension on blood pressure control [24, 43]. A lack of association between hypertension knowledge and self-care behaviour has previously been observed in China’s urban residents, indicating that knowledge may be a better predictor for treatment adherence in hypertensive patients but not for preventive measures in the general community [22, 23]. These differences highlighted that different approaches should be considered when developing and executing blood pressure control programs depending on the goal of prevention or treatment of hypertension. While patient education to increase hypertension knowledge might be effective in controlling blood pressure, implementing the same strategy in a general community for primary prevention might not be efficient [7].

### Health motivations and perceived barriers are main predictors of self-care behaviours for hypertension prevention

It was unsurprising that perceived barriers had the strongest influence on self-care behaviour for hypertension prevention in the present study as it has been consistently reported as the strongest predictor in behaviours for disease prevention, followed by perceived benefits [13, 21, 28]. Interestingly, health motivations, but not perceived benefits strongly influenced hypertension self-care behaviour in the present study. Health motivations have not been evaluated in many studies using the HBM for disease prevention and management, perhaps due to its lack of reported association with behaviour from challenges such as low statistical power and collinearity [20, 28, 44]. A systematic review that examined 41 quantitative research reports and psychometric analyses with health motivation as a variable for self-care behaviours for disease prevention found that motivation was not a significant predictor for over 30% of the studies [44]. In contrast, there is evidence that interventions which aim to enhance an individual’s motivation were effective in promoting self-care behaviours such as controlling alcohol intake and have been implicated in diabetes management [45]. These results indicated an underlying role of motivation in influencing health behaviour for disease prevention and management. Carter and Kulbok (2002) suggested that the lack of influence by motivation on health behaviour may possibly result from ineffective measurement of motivation due to the lack of conceptual clarity in the studies analysed [44]. Health motivations have been described as an overarching theme, influencing HBM predictors including perceived severity, perceived benefits and self-efficacy [45, 46]. Considering the lack of significance of perceived severity, perceived benefits, and self-efficacy to hypertension self-care behaviour in the present study, it is plausible that health motivation is a distinct variable that takes precedence over these HBM variables. On this basis, hypertension prevention interventions should instead focus on minimising barriers against and encouraging motivations for hypertension self-care behaviours through positive reinforcements.

### Barriers for hypertension self-care behaviour

In our study, maintaining a low salt and healthy diet, regular blood pressure check and regular physical activities were the main issues in the self-care behaviour for hypertension prevention although most respondents knew that these behaviours could improve health and prevent disease in general. Our study showed a low frequency of self-care behaviour especially in reducing salt intake, which were consistent with another study which reported that 79% of respondents exceeded the recommended amounts set by local and international health guidelines [47]. Other nation-wide surveys reported that the mean sodium intake in Malaysians was higher than recommended [48–50]. Our study highlighted several barriers of reducing salt intake in the local population such as lack of time for food preparation, limited choices and used to the family diet. These barriers may be attributed to high consumption of heavily salted and fermented foods and sauces such as fish sauce, soy sauce, fish/shrimp pastes in the Malaysian traditional food culture, resulting in inherently high use of sodium in home and commercial cooking [51–53]. It was also reported that nearly 50% of commercially available sauces did not include sodium content on the nutrient information panel, leading to a lack of consumer awareness about sodium contents in food products [52].

In our study, approximately 50–60% of participants practiced regular physical activity and consumed adequate intake of fruits and vegetables. Urbanization is known to cause adverse health effects from reduced energy expenditure due to increased sedentary lifestyle, and shift to a high fat, high energy diet [51, 54]. There appeared to be a lack of healthy food options in commercial areas or work place cafeterias which also include adequate servings of fruits and vegetables, and appeared to be one of the top barriers faced by the community. This may be attributed to increased use of sodium-containing compounds to enhance flavour or improve shelf-life of processed foods, particularly favoured by commercial food vendors [51]. Increased population in the workforce and increased emphasis on education as a result of urbanization may also contribute to the lack of time to exercise or prepare healthier meals at home, leading to increased preference for fast food or processed foods as quicker alternatives (Table 5) [48, 53]. Consistent with literature, feeling ‘lazy’ was another top barrier against regular physical activity among this group, implying that are large proportion of respondents lacked motivation and deemphasized the importance of physical activity [55, 56].

Early stages of hypertension are typically asymptomatic, therefore many are unaware that they have high blood pressure [57, 58]. Regular monitoring of blood pressure is essential for early detection of pre-hypertension and hypertension. In this study, only about 40% of participants always or often check their blood pressure in clinic or at home. The lack of blood pressure checking may be contributing the a high proportion (nearly 50%) of hypertensive patients who were unaware of their hypertension status, as reported in the most recent National Healthy and Morbidity Survey 2019 (NHMS 2019) [3]. Our study highlighted that the ignorant attitude was the greatest barrier to blood pressure monitoring as most of the study participants did not think about checking their blood pressure or thought that it was unnecessary as they were healthy. Furthermore, respondents indicated that blood pressure screening from doctor’s appointments (50%) and free community screening programmes (20%) were top motivators for regular monitoring of blood pressure. This finding suggests that increasing the frequency of blood pressure screening programmes might be an effective strategy to reduce the barrier of blood pressure checking while acting as a cue of action in motivating the public to check their blood pressure. Blood pressure checking should be routinely done as an opportunistic screening for each patient attending to a primary care clinic or during visits to community pharmacies to extend number of screening providers in the community.

### Limitations

The results should be interpreted with caution due to the small sample size, relatively young and middle-aged population and limited to a defined geographical area around the capital city of Malaysia. The cross-sectional nature of the study also indicated that causal inference is limited. Further studies covering different regions or specific communities should be conducted for development of targeted or personalised hypertension prevention interventions. Health motivations, perceived barriers and frequency of hypertension self-care behaviour were measured using the same survey tool (HTN-SCP) based on consistent scoring system with 15 items for each predictor, while the other HBM predictors were evaluated with as few as a single item to minimise the length of the survey. HBM predictors evaluated by only one to three items such as perceived severity, perceived benefits and self-efficacy may not have been measured effectively. Hence, lack of standardization in measurement of each predictor for hypertension self-care behaviour may have introduced some bias in the generated model. Motivators and barriers to smoking cessation were also not evaluated. This poses further opportunities to investigate motivators that encourage non-smoking and uncover barriers to smoking cessation in a relatively well-educated and financially-able population.

## Conclusion

Health motivations (importance of self-care behaviour) and perceived barriers (difficulty in conducting self-care behaviour) were important determinants of preventive hypertension self-care behaviour in the Selangor and Kuala Lumpur community. Perceived barrier had stronger influence on self-care behaviour than perceived motivations. An alarming majority did not practice reduced salt and calorie intake albeit the strong relationship between sodium intake and hypertension development. Frequency of physical activity, fruit and vegetable intake and regular blood pressure monitoring were also inadequate. Cultural, environmental and personal factors contributed to the challenge in practicing these behaviours. Therefore, hypertension prevention intervention or strategies should focus on targeting barriers and motivations of self-care behaviour, particularly barriers against adopting a healthy diet, regular physical activity and blood pressure screenings. More importantly, development and execution of these preventive interventions or strategies require action and cooperation from both government policy makers and the community to reduce the prevalence and burden of hypertension.

## Supporting information

S1 Table Frequency of hypertension self-care behaviour in the Selangor and Kuala Lumpur community. (DOCX)

S2 Table One-sample Kologorov-Smirnov test, Kaiser-Meyer-Olkin and Bartlett’s test and multiple linear regression coefficients on perceived severity, health motivation, perceived benefits, perceived barriers, self-efficacy and hypertension knowledge. (DOCX)

S1 Appendix Survey instrument in English, Bahasa Malaysia and Mandarin. (DOCX)

S1 Checklist Inclusivity in global research. (DOCX)
