# Supplementary material for: Health motivations and perceived barriers are determinants of self-care behaviour for the prevention of hypertension in a Malaysian community
Source: PLoS One. 2022 Dec 7;17(12):e0278761. doi: 10.1371/journal.pone.0278761 (PMC9728916; doi:10.1371/journal.pone.0278761)
Supplement: S1 Table — (DOCX) [file pone.0278761.s001.docx]

| Behaviour | % (N=200) | |
| --- | --- | --- |
|  | always/ often | rarely/ sometimes |
| reduced salt and calorie intake | 18.5 | 81.5 |
| regular blood pressure check | 42.9 | 57.1 |
| regular physical activity | 45.8 | 54.2 |
| > 5 servings of fruits and vegetables | 48.7 | 51.3 |
| moderate consumption of alcohol | 78.2 | 21.8 |
| practice non-smoking | 87.5 | 12.5 |

**S1 Table A.** Frequency of hypertension self-care behaviour in the Selangor and Kuala Lumpur community
