# Supplementary material for: Health motivations and perceived barriers are determinants of self-care behaviour for the prevention of hypertension in a Malaysian community
Source: PLoS One. 2022 Dec 7;17(12):e0278761. doi: 10.1371/journal.pone.0278761 (PMC9728916; doi:10.1371/journal.pone.0278761)

**S3 Table.** One-sample Kologorov-Smirnov test, Kaiser-Meyer-Olkin and Bartlett’s test and multiple linear regression coefficients on perceived severity, health motivation, perceived benefits, perceived barriers, self-efficacy and hypertension knowledge

**Table A** One-sample Kologorov-Smirnov test


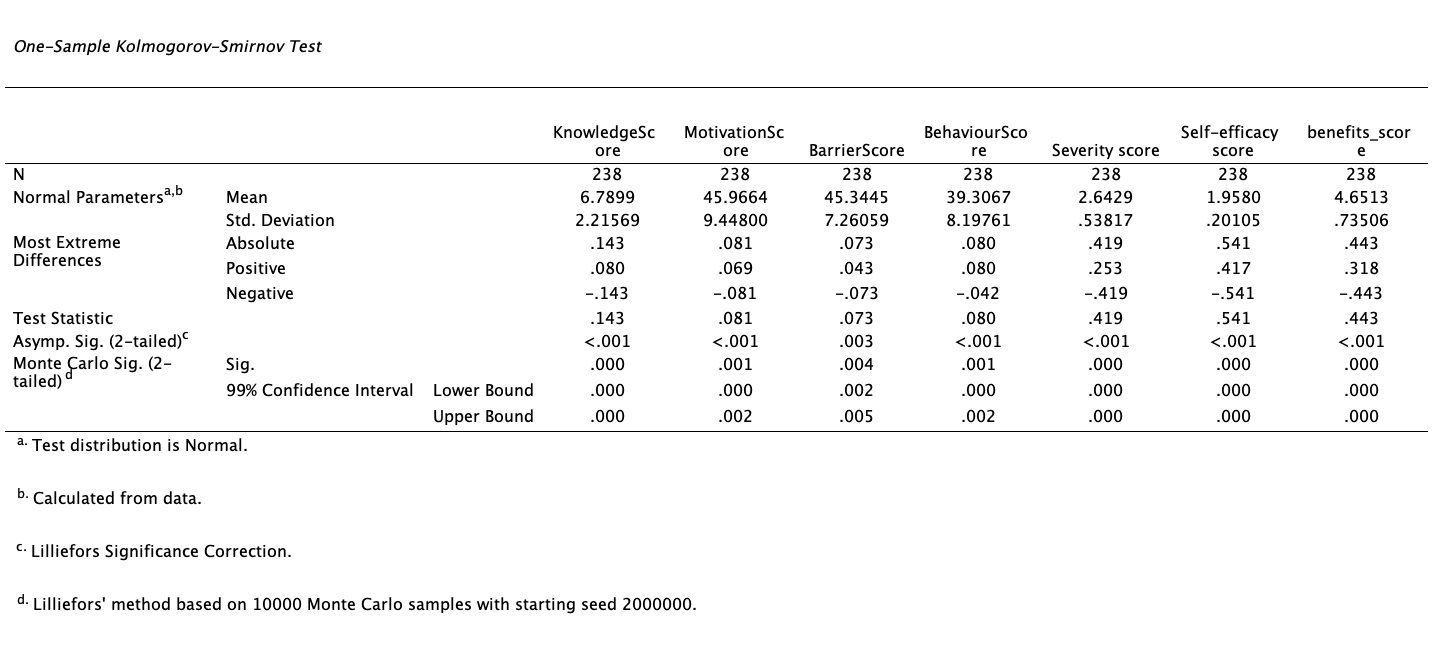


**Table B** Kaiser-Meyer-Olkin and Bartlett’s


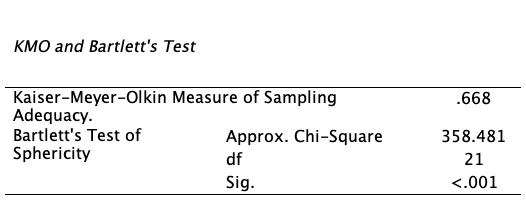


**Table C** Multiple linear regression coefficients and collinearity statistics on perceived severity, health motivation, perceived benefits, perceived barriers, self-efficacy and hypertension knowledge


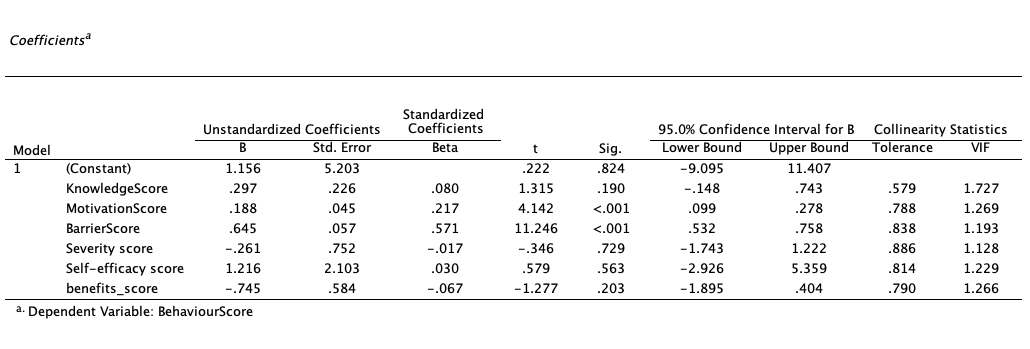

Supplement: S2 Table — (DOCX) [file pone.0278761.s002.docx]
