# Supplementary material for: Health motivations and perceived barriers are determinants of self-care behaviour for the prevention of hypertension in a Malaysian community
Source: PLoS One. 2022 Dec 7;17(12):e0278761. doi: 10.1371/journal.pone.0278761 (PMC9728916; doi:10.1371/journal.pone.0278761)
Supplement: S1 Appendix — (DOCX) [file pone.0278761.s003.docx]

**S2 Appendix.** Survey instrument in English, Bahasa Malaysia and Mandarin

HBM and hypertension self-care behaviour

Start of Block: Participant information sheet

Q1
**Do health beliefs and perceptions towards hypertension influence self-care behaviour?**

Q1
 **Adakah kepercayaan dan tanggapan terhadap hipertensi mempengaruhi tabiat penjagaan diri dalam masyarakat?**

Q1
**个人的高血压观念及看法如何影响自我保健行为？**

Q2 **Participant information sheet**   This is an invitation to take part in a research study on the above title. This information is designed to tell you what it will involve. 
 Your participation is voluntary, and you are free to withdraw at any point before or during the survey. Once you have finished the survey and submitted your answers it is not possible to withdraw the data.

Q2 **Risalah Maklumat Peserta**
   Anda telah dijemput untuk mengambil bahagian dalam sebuah kajian penyelidikan berkenaan dengan tajuk di atas. Risalah ini menjelaskan hal-hal berkenaan penyelidikan tersebut dengan lebih mendalam dan terperinci.

 Penyertaan anda dalam penyelidikan ini adalah secara sukarela. Anda boleh menarik diri daripada soal selidik ini pada bila-bila masa sahaja. Data yang diserahkan pada akhir soal selidik ini tidak boleh ditarik balik.

Q2 **参加者信息表**   本研究者邀请您参加有关上述标题的在线问卷调查。这个参加者信息表是为了通知您这项研究将涉及的内容。   当问卷结束时，所提交的答案并无法撤回。

Q3 **What is this project about?** Hypertension is a major risk factor for cardiovascular disease such as heart attack and stroke. Almost 70% of Malaysians are at risk of elevated high blood pressure. This indicates a large number of individuals that may develop cardiovascular disease in the future if remained uncontrolled. It presents a huge health and economic burden due to direct and indirect costs including life-long treatment on anti-hypertensive medication or reduced productivity. Therefore, there should be an emphasis on prevention of this disease.

 The purpose of this survey is to understand how personal beliefs about health beliefs and views on hypertension influence individual behaviours that might prevent the disease.

Q3 **Apakah tujuan dan latar belakang kajian ini** Hipertensi adalah faktor risiko utama yang boleh menyebabkan penyakit kardiovaskular seperti serangan  jantung dan strok. Hampir 70% rakyat Malaysia menghadapi risiko tekanan darah tinggi. Hal ini menunjukkan bahawa sebilangan besar rakyat Malaysia mungkin menghidapi penyakit kardiovaskular pada masa depan jika tidak dikawal. Hal ini membebankan kesihatan dan ekonomi rakyat dan negara akibat kos langsung dan tidak langsung penyakit ini seperti rawatan sepanjang hayat menggunakan ubat anti-hipertensi atau penurunan produktiviti seseorang individu. Oleh itu, tumpuan terhadap pencegahan penyakit ini perlu diutamakan.

Tujuan soal selidik ini adalah untuk memahami bagaimana kepercayaan dan pandangan seseorang individu terhadap hipertensi mempengaruhi tabiat penjagaan diri yang mungkin dapat mencegah penyakit ini.

Q3 **什么是这项调查的背景与目的？**
 高血压是心血管疾病（如心脏病和中风）的主要危险因素。 几乎70％的马来西亚人民面对血压升高的风险。 这表明一大部分的人民将会患上心血管疾病若不加以控制血压的读数。由于高这些疾病的直接和间接的费用（例终生使用抗高血压药或降低个人的生产力），导致沉重的健康和经济负担。因此，我们应注重预防这种疾病的方式。   本问卷的目是为了理解个人的高血压观念和看法如何影响预防疾病的自我保健行为。

Q5 **Who is being asked to take part?** We would like you to participate in this survey if you are 18 years old and above Malaysian citizen residing in **Selangor and Kuala Lumpur** Have not had any formal diagnosis of hypertension from any medical or healthcare professional

Q5 **Siapakah boleh mengambil bahagian?** Anda boleh mengambil bahagian dalam soal selidik ini jika anda: Berumur 18 tahun ke atas Warganegara Malaysia yang menetap di **Selangor dan Kuala Lumpur** Tidak pernah menerima diagnosis rasmi hipertensi daripada doktor atau mana-mana pakar penjagaan kesihatan

Q5 **谁可以参加这条在线问卷调查？**
 我们希望您参加这条问卷调查，如果您： 18岁以上 居住在雪兰莪和吉隆坡的马来西亚公民 不曾被医生或医护人员正式诊断患有高血压

Q8 **What will I be asked to do?** You will be asked to complete a survey that takes about of **15-20 minutes** to complete. You will be asked about your health beliefs towards hypertension and your current self-care habits. Please answer all questions and answer as honestly as possible.

 When you start the survey, a progress bar will show you how far you are from the end. If you need to pause, that’s fine. You can return to the survey and continue responding to the questions for **up to one week**. After that, your responses will be lost. Please note that you can only participate in this survey only **once.**

Q8 **Apakah tanggungjawab saya sewaktu menyertai kajian ini?** Anda diminta menjawab soal selidik mengenai kepercayaan dan tanggapan kesihatan anda, dan tabiat penjagaan diri. Soal selidik ini mengambil sekitar 15-20 minit untuk diselesaikan. Sila menjawab semua soalan dengan jujur.    Anda akan melihat sebuah '*progress bar'* di bahagian atas halaman ini yang menunjukkan kemajuan anda dalam melengkapkan soal selidik ini. Anda boleh berhenti sebentar dari menjawab soal selidik ini dan kembali lagi untuk bersambung melengkapkannya dalam tempoh **satu minggu**. Jawapan anda akan dipadam seminggu selepas anda memulakan soal selidik ini. Berharap maklum bahawa anda boleh mengambil bahagian dalam soal selidik ini **sekali sahaja.**

Q8 **我参与这条在线问卷调查的责任是什么？** 您将被要求完成用大约15-20分钟的问卷。 此外，问卷将询问您对高血压的健康观念和您本身的自我保健习惯。 请诚实地回答所有的问题。   您应该在页面顶部看到一个问卷进度条以显示您回答问卷的进度。您可暂时停顿回答问卷，并在**一周内**继续完成它。从开始问卷的一周后，您所回复的答案将消失。请注意，您只能回答本问卷**一次**。

Q9 **What will happen to the information I provide?** The data collected will remain anonymous by not requesting any identifiable markers such as name, email address and identification numbers. The data will remain confidential, and only the researcher and the research supervisor will have access to the data. The data will be kept in a password-secured device, with the password only available to the researcher. The research supervisor may see the data upon request. The data will then be kept in the device up to 5 years and for a period of no less than 3 years after the project finishes as per university guidelines.

Q9 **Apakah yang akan berlaku dengan maklumat yang diberikan?**
 Soal selidik ini tidak akan meminta maklumat peribadi seperti nama, emel, dan nombor pengenalan supaya tidak mendedahkan identiti anda. Segala maklumat anda yang diperolehi dalam penyelidikan ini akan disimpan dalam pangkalan data dilindungi dengan kata laluan dan dikendalikan secara sulit. Hanya penyelidik dan penyelia penyelidikan dapat mengakses rekod tersebut. Data yang diperolehi akan disimpan selama tiga tahun hingga 5 tahun setelah kajian ini ditamatkan dan akan dihapuskan secara kekal selepas tempoh penyimpanan mengikut garis panduan universiti

Q9 **我提供的资料会如何被处理？** 为了确保所收集的数据是在匿名的情形，我们不要求任何显示身份的标记，例姓名，电邮地址或生份证。所有的数据将属于机密，唯有研究者和督导们可接入所收集的资料。数据将收存在个由密码保护的电子设备；此外，密码只保持研究者中。根据要求，研究督导可随时查看资料。根据大学方针，这些资料将在指定的电子设备中保存到不少于调查结束的3年至5年。

Q10 **What will you do with the data?** The data will then be analysed for the purposes of the research.

Q10 **Bagaimanakah maklumat tersebut digunakan?**
 Maklumat yang dikumpulkan akan dianalisis untuk tujuan penyelidikan sahaja.

Q10 **研究者怎么利用收集的资料呢？**
您提供的资料将被分析于完成研究的目的。

Q11 An ethics committee has reviewed the proposal and considers that there are no risks to you if you participate in this online survey.

Q11 Jawatankuasa etika telah meluluskan kajian ini dan menganggap bahawa tiada risiko berkaitan dengan kajian ini.

Q11
伦理委员会已批准了这项研究及认为参与这条在线问卷调查并不会带来任何风险。

Q12 **If you are still interested in participating, please click “next” to start the survey**

Q12 **Sekiranya anda berminat untuk menyertai kajian ini, sila klik "seterusnya" untuk memulakan soal selidik**

Q12 **若您有兴趣参加，请单击右下角的按钮以开始问卷**

| Page Break |  |
| --- | --- |

End of Block: Participant information sheet

Start of Block: Participant Consent Form

| 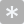 |
| --- |

Q13 I understand that my participation in this study is voluntary and that I am free to withdraw from the study at any time, without giving a reason and without consequence

- Yes
- No

Q13 Saya memahami bahawa penyertaan saya dalam penyelidikan ini adalah secara sukarela dan saya boleh menarik diri daripada soal selidik ini pada bila-bila masa sahaja

- Ya (1)
- Tidak (2)

Q13 我理解参与这条在线问卷调查是出于自愿的，可随时退出及不必提供任何因素

- 是 (1)
- 否 (2)

| 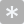 |
| --- |

Q15 I understand that all data are anonymous and that there will not be any connection between the information provided and my identity

- Yes (1)
- No (2)

Q15 Saya memahami bahawa segala maklumat yang diberi akan kekal tanpa nama dan tidak akan mendedahkan identiti saya

- Ya (1)
- Tidak (2)

Q15 我了解所收集的资料都是属于匿名的，此外所提供的资料跟我的身份并没任何关联

- 是 (1)
- 否 (2)

| 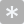 |
| --- |

Q16 I understand that there are no known risks or hazards associated with participating in this study

- Yes (1)
- No (2)

Q16 Saya memahami bahawa tiada risiko yang diketahui berkaitan dengan menyertai kajian penyelidikan ini

- Ya (1)
- Tidak (2)

Q16 我了解参加这条在线问卷调查没有已知的风险或危险

- 是 (1)
- 否 (2)

| 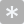 |
| --- |

Q17 I confirm that I have read and understood the above information and that I agree to participate in this study

- Yes (1)
- No (2)

Q17 Saya mengesahkan bahawa saya telah membaca dan memahami maklumat di atas dan bahawa saya bersetuju untuk mengambil bahagian dalam kajian ini

- Ya (1)
- Tidak (2)

Q17 我确认我已阅读并理解上述信息，并同意参加本研究

- 是 (1)
- 否 (2)

Q18 I confirm that I am **over 18 years** of age

- Yes (1)
- No (2)

Q18 Saya mengesahkan bahawa saya berumur **18 tahun dan ke atas**

- Ya (1)
- Tidak (2)

Q18 我确认我已经**超过18岁**

- 是 (1)
- 否 (2)

Q19 I confirm that I am a **Malaysian citizen** **currently living in Selangor or Kuala Lumpur**

- Yes (1)
- No (2)

Q19 Saya mengesahkan bahawa saya seorang **warganergara Malaysia yang menetap di Selangor atau Kuala Lumpur**

- Ya (1)
- Tidak (2)

Q19 我确认我是目前居住在**雪兰莪或吉隆坡的马来西亚公民**

- 是 (1)
- 否 (2)

Q20 I confirm that I have **not** had any formal diagnosis of hypertension by a doctor or medical professional

- Yes (1)
- No (2)

Q20 Saya mengesahkan bahawa saya tidak pernah menerima diagnosis rasmi hipertensi daripada doktor atau mana-mana pakar penjagaan kesihatan

- Ya (1)
- Tidak (2)

Q20 我确认我**不曾**被医生或医护人员正式诊断患有高血压

- 是 (1)
- 否 (2)

Q21 By clicking the button below you indicate that you understand what the study involves and that your answers are anonymous. You agree to take part and you understand that once you click ’submit’ at the end of the questionnaire it will not be possible to withdraw the data.

Q21 Dengan menekan butang di bawah, anda memahami segala yang dilibatkan dalam kajian ini dan jawapan anda adalah secara tanpa nama. Anda bersetuju untuk mengambil bahagian dan memahami bahawa data tidak boleh ditarik balik apabila menekan 'hantar' pada akhir soal selidik ini.

Q21 单击右下角的按钮表示您了解研究内容和了解您提供的答案是匿名的。您同意参与这项研究，并了解一旦在问卷的末尾点击“提交”后就无法撤回您提供的资料。

| Page Break |  |
| --- | --- |

End of Block: Participant Consent Form

Start of Block: Ineligibility

Display This Question:

If I confirm that I am over 18 years of age = No

Or I confirm that I am a Malaysian citizen currently living in Selangor or Kuala Lumpur = No

Or I confirm that I have not had any formal diagnosis of hypertension by a doctor or medical profess... = No

Q51
**Thank you for your interest in participating!**

 Unfortunately, you do not meet to criteria to participate in this survey.

 *Please click the "next" button to end the survey.*

Q51
**Terima kasih atas minat anda untuk menyertai kajian ini!**

 Walaubagaimanapun, anda tidak memenuhi kriteria peserta. 

 *Sila klik butang "seterusnya" untuk menamatkan soal selidik ini.*

Q51
**感谢您的兴趣参加！**

 但是，您不符合这项研究的参加者条件。

 请点击“下一步”结束

Skip To: End of Survey If Thank you for your interest in participating! Unfortunately, you do not meet to criteria to parti... Is Displayed

End of Block: Ineligibility

Start of Block: Demographics

Q22 **Please answer these few questions about yourself**

Q22 **Sila menjawab beberapa soalan mengenai diri anda**

Q22 **请回答以下的问题**

| 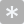 |
| --- |

Q23 Age (years)

________________________________________________________________

Q23 Umur (tahun)

________________________________________________________________

Q23 年龄（年）

________________________________________________________________

Q24 Sex

- Male (1)
- Female (2)

Q24 Jantina

- Lelaki (1)
- Perempuan (2)

Q24 性别

- 男 (1)
- 女 (2)

Q25 Ethinicity

- Malay (1)
- Chinese (2)
- Indian (3)
- Others (4)

Q25 Kumpulan Etnik

- Melayu (1)
- Cina (2)
- India (3)
- Lain-lain (4)

Q25 种族

- 马来人 (1)
- 华人 (2)
- 印度人 (3)
- 其他 (4)

| 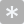 |
| --- |

Q26 Height (cm)

________________________________________________________________

Q26 Ketinggian (cm)

________________________________________________________________

Q26 身高 (cm)

________________________________________________________________

| 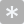 |
| --- |

Q28 Weight (kg)

________________________________________________________________

Q28 Berat badan (kg)

________________________________________________________________

Q28 体重(kg)

________________________________________________________________

Q29 Highest level of education attained

- No formal education received (1)
- Primary School (2)
- Secondary School (3)
- Pre-University (eg. STPM, Matriculation, A-levels, Diploma) (4)
- University (eg. Bachelor's Degree, Master's Degree, PhD) (5)

Q29 Tahap Pendidikan Tertinggi

- Tidak pernah menerima pendidikan formal (1)
- Sekolah rendah (2)
- Sekolah menengah (3)
- Pra-Universiti (cth. STPM, Matrikulasi, A-levels, Diploma) (4)
- Pengajian tinggi (cth. Ijazah Sarjana Muda, Ijazah Sarjana, PhD) (5)

Q29 最高学历

- 没有接受正规教育 (1)
- 小学 (2)
- 中学 (3)
- 大学预科 (例如，STPM, Matriculation, A-levels, Diploma） (4)
- 大学 （例如，学士学位，硕士学位，博士学位） (5)

Q30 Level of monthly household income

- less than RM 999 (1)
- RM 1000 – RM 3999 (2)
- RM 4000 – RM 9500 (3)
- more than RM 9500 (4)

Q30 Pendapatan isi rumah bulanan

- kurang daripada RM 999 (1)
- RM 1000 – RM 3999 (2)
- RM 4000 – RM 9500 (3)
- lebih daripada RM 9500 (4)

Q30 每月家庭收入水平

- 少过 RM 999 (1)
- RM 1000 – RM 3999 (2)
- RM 4000 – RM 9500 (3)
- 多过 RM 9500 (4)

Q31 Has anyone in your family had any of the following?

|  | Yes (1) | No (2) | I don't know (3) |
| --- | --- | --- | --- |
| ⊗High blood pressure (1) |  |  |  |
| ⊗Diabetes (2) |  |  |  |
| ⊗High cholesterol (3) |  |  |  |
| ⊗Kidney disease (4) |  |  |  |
| ⊗Heart disease (eg heart attack) (5) |  |  |  |
| ⊗Stroke (6) |  |  |  |

Q31 Adakah ahli keluarga anda mengalami keadaan berikut?

|  | Ya (1) | Tidak (2) | Tidak tahu (3) |
| --- | --- | --- | --- |
| ⊗Tekanan darah tinggi (1) |  |  |  |
| ⊗Kencing manis (2) |  |  |  |
| ⊗Kolesterol tinggi (3) |  |  |  |
| ⊗Penyakit ginjal (4) |  |  |  |
| ⊗Penyakit jantung (cth. Serangan jantung) (5) |  |  |  |
| ⊗Strok (6) |  |  |  |

Q31 您家中有谁曾经患上以下的疾病吗？

|  | 有 (1) | 没有 (2) | 我不知道 (3) |
| --- | --- | --- | --- |
| ⊗高血压 (high blood pressure) (1) |  |  |  |
| ⊗糖尿病 (diabetes) (2) |  |  |  |
| ⊗高胆固醇 （high cholesterol) (3) |  |  |  |
| ⊗肾脏疾病 (kidney disease) (4) |  |  |  |
| ⊗心脏病 (heart disease/heart attack) (5) |  |  |  |
| ⊗中风 （stroke) (6) |  |  |  |

| Page Break |  |
| --- | --- |

End of Block: Demographics

Start of Block: Knowledge

Q32 **Listed below are some questions to assess your knowledge of hypertension**

Q32 **Berikut adalah beberapa soalan untuk menilai pengetahuan anda mengenai hipertensi**

Q32 **以下是一些有关高血压认识的问题**

Q33 What does the term hypertension mean?

- High blood pressure (1)
- High stress level/ tension (2)
- Nervous condition (3)
- High blood sugar (4)
- Overactivity (5)
- Don't know (6)

Q33 Apakah maksud "hipertensi"?

- Tekanan darah itnggi (1)
- Tahap tekanan/ ketegangan tinggi (2)
- Keadaan saraf (3)
- Gula darah tinggi (4)
- Hiperaktif (5)
- Tidak tahu (6)

Q33 "高血压"是什么意思?

- 血压升高的状态 (1)
- 压力过高 (2)
- 紧张状态 (3)
- 高血糖 (4)
- 过于劳动 (5)
- 我不知道 (6)

Q34 How dangerous is hypertension to your health?

- Extremely (1)
- Somewhat (2)
- Not at all (3)
- Don't know (4)

Q34 Adakah hipertensi berbahaya untuk kesihatan anda?

- Sangat bahaya (1)
- Agak bahaya (2)
- Tidak bahaya (3)
- Tidak tahu (4)

Q34 高血压对个人的身体健康带来多大的危害？

- 非常 (1)
- 有些危害 (2)
- 完全没有 (3)
- 我不知道 (4)

Q35 Would lowering high blood pressure improve a person's health?

- Yes (1)
- No (2)
- Somewhat (3)
- Don't know (4)

Q35 Bolehkah seseorang meningkatkan kesihatannya dengan menurunkan tekanan darah tinggi?

- Ya (1)
- Tidak (2)
- Agak boleh (3)
- Tidak tahu (4)

Q35 降低血压会不会对人的健康有所改善？

- 会 (1)
- 不会 (2)
- 或许 (3)
- 我不知道 (4)

Q36 *130/80mmHg is an example of a blood pressure reading*
 What do the two numbers reported for blood pressure mean?
Note: you can leave it blank if you do not know the answers

- Top number (1) ________________________________________________
- Bottom number (2) ________________________________________________

Q36 *130/80 mmHg adalah contoh bacaan tekanan darah* Apakah maksud dua nombor yang dilaporkan untuk tekanan darah? *Nota: anda boleh biarkan kosong jika tidak tahu jawapan*

- Nombor atas (1) ________________________________________________
- Nombor bawah (2) ________________________________________________

Q36 *130/80mmHg 是血压读数的例子 （2个血压读数）*
  这两个数字代表什么？ *注：若不会回答，您可以将其留空*

- 上面数字 (1) ________________________________________________
- 下面数字 (2) ________________________________________________

Q39 What should the normal blood pressure levels be?

Q39 Apakah tahap tekanan darah yang biasa?

Q39 正常的血压读数因该是多少？

Q41 Top number:

- Less than 140mmHg (4)
- More than 140mmHg (5)
- Don't know (6)

Q41 Nombor atas:

- Kurang daripada 140mmHg (4)
- Lebih daripada 140mmHg (5)
- Tidak tahu (6)

Q41 上面数字:

- 少于 140mmHg (4)
- 多于 140mmHg (5)
- 我不知道 (6)

Q50 Bottom number:

- Less than 90mmHg (4)
- More than 90mmHg (5)
- Don't know (6)

Q50 Nombor bawah:

- Kurang daripada 90mmHg (4)
- Lebih daripada 90mmHg (5)
- Tidak tahu (6)

Q50 下面数字:

- 少于 90mmHg (4)
- 多于 90mmHg (5)
- 我不知道 (6)

Q42 Which measure(s) is (are) more important?

- Top (4)
- Bottom (5)
- Both (6)

Q42 Ukuran darah tinggi manakah yang lebih penting?

- Nombor atas (4)
- Nombor bawah (5)
- Kedua-dua nombor (6)

Q42 哪个读数数字比较重要?

- 上面数字 (4)
- 下面数字 (5)
- 两个数字 (6)

Q43 Can people do things to lower their blood pressure?

- Yes (1)
- No (2)
- Don't know (3)

Q43 Bolehkah seseorang melakukan sesuatu untuk menurunkan tekanan darah diri?

- Ya (1)
- Tidak (2)
- Tidak tahu (3)

Q43 人们可不可以做些事情来降低自己的血压水平？

- 是 (1)
- 否 (2)
- 我不知道 (3)

Q44 Can lowering blood pressure even a little bit improve health?

- Yes (1)
- No (2)
- Don't know (3)

Q44 Bolehkah menurunkan tekanan darah walaupun sedikit, meningkatkan kesihatan seseorang?

- Ya (1)
- Tidak (2)
- Tidak tahu (3)

Q44 甚至少许地降低血压水平是否能帮助健康有所改善？

- 是 (1)
- 否 (2)
- 我不知道 (3)

| Page Break |  |
| --- | --- |

End of Block: Knowledge

Start of Block: Self-care behaviour

Q45 **This section contains 15 statements about what you can do to prevent or control hypertension. For each of the 15 statements, please respond “how important it is for you”, “how difficult it is for you” and “how often” you carry out these actions or behaviours.**
 
*Your honest responses are appreciated*

Q45 **Bahagian ini akan mengandungi 15 saranan lazim penjagaan diri untuk mencegah dan mengawal hipertensi. Untuk setiap 15 saranan penjagaan diri tersebut, sila nyatakan “berapa penting”, “berapa sukar” dan “berapa sering” anda melakukan saranan ini.**

 *Jawapan dengan jujur amat dihargai*

Q45 这个部分将列出**15**个陈述。这些陈述是预防和控制高血压的自我保健常见建议。对于这15个陈述，请回答您对进行这些自我保健行为的重要性，难易度和经常性。 

 *感谢您诚实的回应。*

| Page Break |  |
| --- | --- |

Q48 In general, how **important** is it for you to do the following?

|  | Very Important (1) | Important (2) | Fairly important (3) | Not important (4) |
| --- | --- | --- | --- | --- |
| Take part in regular physical activity (eg. 30 minutes of walking 4-5 times per week (1) |  |  |  |  |
| Eat less processed foods (e.g., canned or frozen foods, half-cooked meat) (2) |  |  |  |  |
| Read nutrition facts label to check information on sodium content (3) |  |  |  |  |
| Read nutrition label to check info on saturated (eg red meat, butter) fat and transfat (eg shortening, lard) (4) |  |  |  |  |
| Replace traditional high-salt foods (eg. Canned soup, instant noodles) with low salt products (eg. Homemade soups, fresh vegetables) (5) |  |  |  |  |
| Limit the use of high-salt condiments (eg ketchup) (6) |  |  |  |  |
| Eat less than 1 teaspoon of table salt per day 6g salt = 1 bowl Mee Soto or 1 bowl Mee Rebus or 2 plates of chicken rice (7) |  |  |  |  |
| Eat less foods that are high in saturated (eg red meat, butter) fat and transfat (eg shortening, lard) (8) |  |  |  |  |
| Replace traditional high-fat foods (eg deep fried chicken) with low-fat products (eg baked chicken) (9) |  |  |  |  |
| Limit total calorie intake from fat (less than 65grams) daily. 65g fat = 3 plates of Char Kway Teow or 3 plates of Mee Rebus or 2 bowls of Laksa or 3 plates of fried noodles or 3 plates of chicken rice (10) |  |  |  |  |
| Use grill, bake or steam instead of frying when cooking (11) |  |  |  |  |
| Eat 5 or more servings of fruits and vegetables daily (12) |  |  |  |  |
| Practice moderation in drinking alcohol (2 glasses or less for men; 1 glass or less for women) (13) |  |  |  |  |
| Practice non-smoking (14) |  |  |  |  |
| Check your blood pressure at home or at a clinic (at least once a year) (15) |  |  |  |  |

Q48 Secara umum, berapa **pentingkah**ia kepada anda untuk melakukan perkara berikut?

|  | Sangat penting (1) | Penting (2) | Agak penting (3) | Tidak penting (4) |
| --- | --- | --- | --- | --- |
| Mengambil bahagian dalam aktiviti fizikal secara tetap (cth. 30 minit berjalan kaki, 4-5 kali seminggu) (1) |  |  |  |  |
| Kurangkan makan makanan yang diproses seperti (cth. makanan tin atau sejuk beku, daging separuh masak) (2) |  |  |  |  |
| Membaca label fakta pemakanan untuk memeriksa maklumat tentang kandungan natrium (3) |  |  |  |  |
| Membaca label fakta pemakanan untuk memeriksa maklumat tentang lemak tepu (cth. mentega, daging merah) dan lemak trans (cth. lelemak) (4) |  |  |  |  |
| Menggantikan makanan tradisional yang mempunyai kandungan garam yang tinggi (cth. sup dalam tin, mi segera) dengan produk yang mempunyai kandungan garam yang rendah (cth. sup yang dimasak sendiri di rumah, sayur-sayuran segar) (5) |  |  |  |  |
| Mengehadkan penggunaan perencah yang mempunyai kandungan garam yang tinggi (cth. sos tomato) (6) |  |  |  |  |
| Makan kurang daripada 1 sudu kecil garam setiap hari (6 gram)? 6g garam = 1 mangkuk Mee Soto atau 1 mangkuk Mee Rebus atau 2 pinggan Nasi Ayam (7) |  |  |  |  |
| Kurangkan makan makanan yang mengandungi lemak tepu yang tinggi (cth. daging merah, mentega) dan lemak trans (cth. lelemak) (8) |  |  |  |  |
| Menggantikan makanan tradisional yang mengandungi banyak lemak (cth. ayam goreng) dengan produk rendah lemak (cth. ayam bakar) (9) |  |  |  |  |
| Mengehadkan jumlah pengambilan kalori daripada lemak (kurang daripada 65 gram) setiap hari? 65g lemak = 3 pinggan Char Kway Teow atau 3 pinggan Mee Rebus atau 2 mangkuk Laksa atau 3 pinggan Mee Goreng atau 3 pinggan Nasi Ayam (10) |  |  |  |  |
| Gunakan cara memanggang, membakar atau mengukus daripada menggoreng apabila anda memasak (11) |  |  |  |  |
| Makan 5 atau lebih hidangan buah-buahan dan sayur-sayuran setiap hari (12) |  |  |  |  |
| Mengamalkan kesederhanaan dalam pengambilan alkohol setiap hari (2 gelas atau kurang untuk lelaki; 1 gelas atau kurang untuk wanita) (13) |  |  |  |  |
| Mengamalkan tabiat tidak merokok (14) |  |  |  |  |
| Memeriksa tekanan darah anda di rumah ataupun di klinik (sekurang-kurangnya sekali setahun) (15) |  |  |  |  |

Q48 一般来说，进行以下的事件对您而言有**多重要**？

|  | 非常重要 (1) | 重要 (2) | 相当重要 (3) | 不重要 (4) |
| --- | --- | --- | --- | --- |
| 经常运动经常运动（例：散步30 分钟每个星期4-5 次） (1) |  |  |  |  |
| 少吃加工食品（例：罐头食品，冷冻食品，半熟肉类） (2) |  |  |  |  |
| 阅读营养标签来检查盐的含量 (3) |  |  |  |  |
| 阅读营养标签来检查饱和脂肪（例：红肉和奶油）和反式脂肪（例：起酥油和猪油）的含量 (4) |  |  |  |  |
| 用含盐量低食品（例: 自制汤，新鲜蔬菜）来替代含盐量高的食品（例：罐头汤，快熟面 (5) |  |  |  |  |
| 限制使用高盐调味品（例：番茄酱） (6) |  |  |  |  |
| 每日食用不多于1茶匙的食盐。6克食盐=1 碗 Mee Soto 或1 碗 Mee Rebus 或2 碟鸡饭 (7) |  |  |  |  |
| 少吃高饱和脂肪（例：红肉和奶油）和反式脂肪（例：起酥油和猪油）含量的食品 (8) |  |  |  |  |
| 用低脂肪的食品（例：烤鸡）来替代高脂肪的食品 （例：炸鸡） (9) |  |  |  |  |
| 限制每日的脂肪摄入的总卡路里（不多于65克）。65克脂肪=3 碟炒粿条或3 碗Mee Rebus 或2 碗Laksa 或3 碟炒面或3 碟鸡饭 (10) |  |  |  |  |
| 烹饪时用烤，烘或蒸来替代炸 (11) |  |  |  |  |
| 每日吃至少5份蔬菜水果 (12) |  |  |  |  |
| 适当的饮酒（男人不多于2杯，女人不多于1杯） (13) |  |  |  |  |
| 不抽烟 (14) |  |  |  |  |
| 在家里或诊所检查血压（每年至少一次） (15) |  |  |  |  |

| Page Break |  |
| --- | --- |

Q53 In general, how **difficult** is it for you to do the following self-care habits regularly?

|  | Easy (1) | Fairly easy (2) | Fairly difficult (3) | Very difficult (4) |
| --- | --- | --- | --- | --- |
| Take part in regular physical activity (eg. 30 minutes of walking 4-5 times per week (1) |  |  |  |  |
| Eat less processed foods (e.g., canned or frozen foods, half-cooked meat) (2) |  |  |  |  |
| Read nutrition facts label to check information on sodium content (3) |  |  |  |  |
| Read nutrition label to check info on saturated (eg red meat, butter) fat and transfat (eg shortening, lard) (4) |  |  |  |  |
| Replace traditional high-salt foods (eg. Canned soup, instant noodles) with low salt products (eg. Homemade soups, fresh vegetables) (5) |  |  |  |  |
| Limit the use of high-salt condiments (eg ketchup) (6) |  |  |  |  |
| Eat less than 1 teaspoon of table salt per day 6g salt = 1 bowl Mee Soto or 1 bowl Mee Rebus or 2 plates of chicken rice (7) |  |  |  |  |
| Eat less foods that are high in saturated (eg red meat, butter) fat and transfat (eg shortening, lard) (8) |  |  |  |  |
| Replace traditional high-fat foods (eg deep fried chicken) with low-fat products (eg baked chicken) (9) |  |  |  |  |
| Limit total calorie intake from fat (less than 65grams) daily. 65g fat = 3 plates of Char Kway Teow or 3 plates of Mee Rebus or 2 bowls of Laksa or 3 plates of fried noodles or 3 plates of chicken rice (10) |  |  |  |  |
| Use grill, bake or steam instead of frying when cooking (11) |  |  |  |  |
| Eat 5 or more servings of fruits and vegetables daily (12) |  |  |  |  |
| Practice moderation in drinking alcohol (2 glasses or less for men; 1 glass or less for women) (13) |  |  |  |  |
| Practice non-smoking (14) |  |  |  |  |
| Check your blood pressure at home or at a clinic (at least once a year) (15) |  |  |  |  |

Q53 Secara umum, berapa **sukarkah** ia kepada anda untuk melakukan perkara berikut?

|  | Tidak sukar (1) | Agak sukar (2) | Sukar (3) | Sangat sukar (4) |
| --- | --- | --- | --- | --- |
| Mengambil bahagian dalam aktiviti fizikal secara tetap (cth. 30 minit berjalan kaki, 4-5 kali seminggu) (1) |  |  |  |  |
| Kurangkan makan makanan yang diproses seperti (cth. makanan tin atau sejuk beku, daging separuh masak) (2) |  |  |  |  |
| Membaca label fakta pemakanan untuk memeriksa maklumat tentang kandungan natrium (3) |  |  |  |  |
| Membaca label fakta pemakanan untuk memeriksa maklumat tentang lemak tepu (cth. mentega, daging merah) dan lemak trans (cth. lelemak) (4) |  |  |  |  |
| Menggantikan makanan tradisional yang mempunyai kandungan garam yang tinggi (cth. sup dalam tin, mi segera) dengan produk yang mempunyai kandungan garam yang rendah (cth. sup yang dimasak sendiri di rumah, sayur-sayuran segar) (5) |  |  |  |  |
| Mengehadkan penggunaan perencah yang mempunyai kandungan garam yang tinggi (cth. sos tomato) (6) |  |  |  |  |
| Makan kurang daripada 1 sudu kecil garam setiap hari (6 gram)? 6g garam = 1 mangkuk Mee Soto atau 1 mangkuk Mee Rebus atau 2 pinggan Nasi Ayam (7) |  |  |  |  |
| Kurangkan makan makanan yang mengandungi lemak tepu yang tinggi (cth. daging merah, mentega) dan lemak trans (cth. lelemak) (8) |  |  |  |  |
| Menggantikan makanan tradisional yang mengandungi banyak lemak (cth. ayam goreng) dengan produk rendah lemak (cth. ayam bakar) (9) |  |  |  |  |
| Mengehadkan jumlah pengambilan kalori daripada lemak (kurang daripada 65 gram) setiap hari? 65g lemak = 3 pinggan Char Kway Teow atau 3 pinggan Mee Rebus atau 2 mangkuk Laksa atau 3 pinggan Mee Goreng atau 3 pinggan Nasi Ayam (10) |  |  |  |  |
| Gunakan cara memanggang, membakar atau mengukus daripada menggoreng apabila anda memasak (11) |  |  |  |  |
| Makan 5 atau lebih hidangan buah-buahan dan sayur-sayuran setiap hari (12) |  |  |  |  |
| Mengamalkan kesederhanaan dalam pengambilan alkohol setiap hari (2 gelas atau kurang untuk lelaki; 1 gelas atau kurang untuk wanita) (13) |  |  |  |  |
| Mengamalkan tabiat tidak merokok (14) |  |  |  |  |
| Memeriksa tekanan darah anda di rumah ataupun di klinik (sekurang-kurangnya sekali setahun) (15) |  |  |  |  |

Q53 一般来说，经常执行以下的事件对您而言有**多困难**？

|  | 非常容易 (1) | 容易 (2) | 困难 (3) | 非常困难 (4) |
| --- | --- | --- | --- | --- |
| 经常运动（例：散步30 分钟每个星期4-5 次） (1) |  |  |  |  |
| 少吃加工食品（例：罐头食品，冷冻食品，半熟肉类） (2) |  |  |  |  |
| 阅读营养标签来检查盐的含量 (3) |  |  |  |  |
| 阅读营养标签来检查饱和脂肪（例：红肉和奶油）和反式脂肪（例：起酥油和猪油）的含量 (4) |  |  |  |  |
| 用含盐量低食品（例：自制汤，新鲜蔬菜）来替代含盐量高的传统食品（例：罐头汤，快熟面） (5) |  |  |  |  |
| 限制使用高盐调味品（例：番茄酱） (6) |  |  |  |  |
| 每日食用不多于1茶匙的食盐。6克食盐=1 碗 Mee Soto 或1 碗 Mee Rebus 或2 碟鸡饭 (7) |  |  |  |  |
| 少吃高饱和脂肪（例：红肉和奶油）和反式脂肪（例：起酥油和猪油）含量的食品 (8) |  |  |  |  |
| 用低脂肪的食品（例：烤鸡）来替代高脂肪的食品 （例：炸鸡） (9) |  |  |  |  |
| 限制每日的脂肪摄入的总卡路里（不多于65克） 65克脂肪=3 碟炒粿条或3 碗Mee Rebus 或2 碗Laksa 或3 碟炒面或3 碟鸡饭 (10) |  |  |  |  |
| 烹饪时用烤，烘或蒸来替代炸 (11) |  |  |  |  |
| 每日吃至少5份蔬菜水果 (12) |  |  |  |  |
| 适当的饮酒（男人不多于2杯，女人不多于1杯） (13) |  |  |  |  |
| 不抽烟 (14) |  |  |  |  |
| 在家里或诊所检查血压（每年至少一次） (15) |  |  |  |  |

| Page Break |  |
| --- | --- |

Q54 How **often** do you do the following?

|  | Always (1) | Often (2) | Sometimes (3) | Rarely/never (4) |
| --- | --- | --- | --- | --- |
| Take part in regular physical activity (eg. 30 minutes of walking 4-5 times per week (1) |  |  |  |  |
| Eat less processed foods (e.g., canned or frozen foods, half-cooked meat) (2) |  |  |  |  |
| Read nutrition facts label to check information on sodium content (3) |  |  |  |  |
| Read nutrition label to check info on saturated (eg red meat, butter) fat and transfat (eg shortening, lard) (4) |  |  |  |  |
| Replace traditional high-salt foods (eg. Canned soup, instant noodles) with low salt products (eg. Homemade soups, fresh vegetables) (5) |  |  |  |  |
| Limit the use of high-salt condiments (eg ketchup) (6) |  |  |  |  |
| Eat less than 1 teaspoon of table salt per day 6g salt = 1 bowl Mee Soto or 1 bowl Mee Rebus or 2 plates of chicken rice (7) |  |  |  |  |
| Eat less foods that are high in saturated (eg red meat, butter) fat and transfat (eg shortening, lard) (8) |  |  |  |  |
| Replace traditional high-fat foods (eg deep fried chicken) with low-fat products (eg baked chicken) (9) |  |  |  |  |
| Limit total calorie intake from fat (less than 65grams) daily. 65g fat = 3 plates of Char Kway Teow or 3 plates of Mee Rebus or 2 bowls of Laksa or 3 plates of fried noodles or 3 plates of chicken rice (10) |  |  |  |  |
| Use grill, bake or steam instead of frying when cooking (11) |  |  |  |  |
| Eat 5 or more servings of fruits and vegetables daily (12) |  |  |  |  |
| Practice moderation in drinking alcohol (2 glasses or less for men; 1 glass or less for women) (13) |  |  |  |  |
| Practice non-smoking (14) |  |  |  |  |
| Check your blood pressure at home or at a clinic (at least once a year) (15) |  |  |  |  |

Q54 Berapa **kerapkah** anda melakukan perkara berikut?

|  | Sentiasa (1) | Kerap kali (2) | Kadangkala (3) | Jarang-jarang/Tidak pernah (4) |
| --- | --- | --- | --- | --- |
| Mengambil bahagian dalam aktiviti fizikal secara tetap (cth. 30 minit berjalan kaki, 4-5 kali seminggu) (1) |  |  |  |  |
| Kurangkan makan makanan yang diproses seperti (cth. makanan tin atau sejuk beku, daging separuh masak) (2) |  |  |  |  |
| Membaca label fakta pemakanan untuk memeriksa maklumat tentang kandungan natrium (3) |  |  |  |  |
| Membaca label fakta pemakanan untuk memeriksa maklumat tentang lemak tepu (cth. mentega, daging merah) dan lemak trans (cth. lelemak) (4) |  |  |  |  |
| Menggantikan makanan tradisional yang mempunyai kandungan garam yang tinggi (cth. sup dalam tin, mi segera) dengan produk yang mempunyai kandungan garam yang rendah (cth. sup yang dimasak sendiri di rumah, sayur-sayuran segar) (5) |  |  |  |  |
| Mengehadkan penggunaan perencah yang mempunyai kandungan garam yang tinggi (cth. sos tomato) (6) |  |  |  |  |
| Makan kurang daripada 1 sudu kecil garam setiap hari (6 gram)? 6g garam = 1 mangkuk Mee Soto atau 1 mangkuk Mee Rebus atau 2 pinggan Nasi Ayam (7) |  |  |  |  |
| Kurangkan makan makanan yang mengandungi lemak tepu yang tinggi (cth. daging merah, mentega) dan lemak trans (cth. lelemak) (8) |  |  |  |  |
| Menggantikan makanan tradisional yang mengandungi banyak lemak (cth. ayam goreng) dengan produk rendah lemak (cth. ayam bakar) (9) |  |  |  |  |
| Mengehadkan jumlah pengambilan kalori daripada lemak (kurang daripada 65 gram) setiap hari? 65g lemak = 3 pinggan Char Kway Teow atau 3 pinggan Mee Rebus atau 2 mangkuk Laksa atau 3 pinggan Mee Goreng atau 3 pinggan Nasi Ayam (10) |  |  |  |  |
| Gunakan cara memanggang, membakar atau mengukus daripada menggoreng apabila anda memasak (11) |  |  |  |  |
| Makan 5 atau lebih hidangan buah-buahan dan sayur-sayuran setiap hari (12) |  |  |  |  |
| Mengamalkan kesederhanaan dalam pengambilan alkohol setiap hari (2 gelas atau kurang untuk lelaki; 1 gelas atau kurang untuk wanita) (13) |  |  |  |  |
| Mengamalkan tabiat tidak merokok (14) |  |  |  |  |
| Memeriksa tekanan darah anda di rumah ataupun di klinik (sekurang-kurangnya sekali setahun) (15) |  |  |  |  |

Q54 您有**多经常**进行以下的事件？

|  | 总是 (1) | 时常 (2) | 有时 (3) | 很少/从来没有 (4) |
| --- | --- | --- | --- | --- |
| 经常运动（例：散步30 分钟每个星期4-5 次） (1) |  |  |  |  |
| 少吃加工食品（例：罐头食品，冷冻食品，半熟肉类） (2) |  |  |  |  |
| 阅读营养标签来检查盐的含量 (3) |  |  |  |  |
| 阅读营养标签来检查饱和脂肪（例：红肉和奶油）和反式脂肪（例：起酥油和猪油）的含量 (4) |  |  |  |  |
| 用含盐量低食品（例：自制汤，新鲜蔬菜）来替代含盐量高的传统食品（例：罐头汤，快熟面） (5) |  |  |  |  |
| 限制使用高盐调味品（例：番茄酱） (6) |  |  |  |  |
| 每日食用不多于1茶匙的食盐。6克食盐=1 碗 Mee Soto 或1 碗 Mee Rebus 或2 碟鸡饭 (7) |  |  |  |  |
| 少吃高饱和脂肪（例：红肉和奶油）和反式脂肪（例：起酥油和猪油）含量的食品 (8) |  |  |  |  |
| 用低脂肪的食品（例：烤鸡）来替代高脂肪的食品 （例：炸鸡） (9) |  |  |  |  |
| 限制每日的脂肪摄入的总卡路里（不多于65克） 65克脂肪=3 碟炒粿条或3 碗Mee Rebus 或2 碗Laksa 或3 碟炒面或3 碟鸡饭 (10) |  |  |  |  |
| 烹饪时用烤，烘或蒸来替代炸 (11) |  |  |  |  |
| 每日吃至少5份蔬菜水果 (12) |  |  |  |  |
| 适当的饮酒（男人不多于2杯，女人不多于1杯） (13) |  |  |  |  |
| 不抽烟 (14) |  |  |  |  |
| 在家里或诊所检查血压（每年至少一次） (15) |  |  |  |  |

| Page Break |  |
| --- | --- |

End of Block: Self-care behaviour

Start of Block: Factors

Q50    Based on your current self-care behaviour we would like to know what **motivates** or **prevents** you from practicing these behaviours. The previous 15 recommendations have been grouped into **five separate categories** – physical activity, salt and calorie intake, fruit and vegetable intake, alcohol intake, and blood pressure screening.

Q50 Berdasarkan tingkah laku tabiat penjagaan diri anda sekarang, kami ingin mengetahui apakah **mendorong** atau **menghalang** anda daripada mengamalkan tabiat ini. Kesemua 15 saranan lazim penjagaan diri telah dibahagikan kepada **lima kategori yang berasingan** iaitu aktiviti fizikal, pengambilan garam dan kalori, pengambilan buah dan sayur, pengambilan alkohol, dan pemeriksaan tekanan darah.

Q50 根据您目前的自我保健行为，我们想知道什么**促使**和**阻止**您进行这些习惯。前一部分的15个建议已被**分为5组**，分别是，体能运动，盐和卡路里的摄取量，蔬菜水果的摄取量，饮酒量以及血压筛查。

| Page Break |  |
| --- | --- |

| 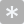 |
| --- |

Q52 In the previous section, you have selected '*Always*', '*Often*', '*Sometimes*', or '*Rarely/never*' for the following self-care behaviour:

 **Take part in regular physical activity (eg. 30 minutes of walking 4-5 times per week)**
   If you **often or always** do so, what motivates you to engage in the behaviour? OR
 If you **sometimes, rarely or never** do so, what prevents you from always engaging in the behaviour?
 *Please select a maximum of three* factors that influences how often you carry out the behaviour the most.

- exercising helps me relieve stress (1)
- exercising can help to improve and maintain health (2)
- motivation from friends and family (3)
- to keep fit (4)
- to keep slim for beauty purposes (5)
- no time (irregular work hours/ busy lifestyle) (6)
- lack of motivation/ lazy (7)
- lack of safe/ affordable place to exercise (8)
- Others (Please specify) (9) ________________________________________________

Q52 Dalam bahagian sebelum ini, anda telah memilih 'Sentiasa', 'Kerap kali‘, 'Kadangkala', atau ’Jarang-jarang/ tidak pernah' untuk tabiat penjagaan diri yang berikut:

 **Mengambil bahagian dalam aktiviti fizikal secara tetap (cth. 30 minit berjalan kaki, 4-5 kali seminggu)?**

 Jika **sentiasa atau kerap** melakukannya, apakah mendorong anda melibatkan diri dalam tabiat tersebut? ATAU
 Jika **kadangkala, jarang-jarang atau tidak pernah** melakukannya, apakah menghalang anda dari sentiasa melibatkan diri dalam tabiat tersebut?
 *Sila pilih maksimum tiga faktor yang paling mempengaruhi tingkah laku anda.*

- bersenam membantu saya menghilangkan tekanan (1)
- bersenam dapat membantu meningkatkan dan menjaga kesihatan (2)
- motivasi daripada rakan dan keluarga (3)
- untuk mejaga kecergasan badan (4)
- menjaga kelihatan badan untuk tujuan kecantikkan (5)
- tiada masa (waktu kerja yang tidak teratur/ gaya hidup sibuk) (6)
- kurang motivasi/ malas (7)
- kekurangan tempat bersenam yang selamat / berpatutan (8)
- Lain-lain (Sila nyatakan) (9) ________________________________________________

Q52 在前一部分，您为以下的自我保健行为选择了“总是”，“时常”，“有时”或“很少/从来没有”：
   **经常运动（如：散步30 分钟每个星期4-5 次）**

 若您**总是或时常**进行，是什么促进您这样做？或 若您**有时，很少或从来没有**进行，是什么阻止您时常这样做？

 *请选择不多于****3*** *最适合您的原因。若以下的原因都不适合请另行写下自己的原因。*

- 运动帮助纾解压力 (1)
- 运动帮助改善和维持健康 (2)
- 动力来自于朋友和家人 (3)
- 保持身材 (4)
- 为了美感而保持身材 (5)
- 缺乏时间（不规律的工作时间/忙碌生活） (6)
- 缺乏动力/懒惰 (7)
- 缺乏安全/实惠的运动场所 (8)
- 其他(请明确说明) (9) ________________________________________________

| Page Break |  |
| --- | --- |

| 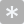 |
| --- |

Q54 In the previous section, you have selected '*Always*', '*Often*', '*Sometimes*', or '*Rarely/never*' for the following self-care behaviour:
 Limit your intake of salt, and calorie intake from fat, and fried foods If you **often or always** do so, what motivates you to engage in the behaviour? OR
 If you **sometimes, rarely or never** do so, what prevents you from always engaging in the behaviour?
 *Please select a maximum of three* factors that influences your behaviour the most.

- maintain health (1)
- to lose weight (2)
- prevent various diseases (3)
- limited food choices when eating out (4)
- healthy foods are more expensive (5)
- no time to prepare healthy foods, instant meals or fast food is a quicker alternative (6)
- sharing a diet with my family is challenging (7)
- I prefer the taste of salty/ fried foods (8)
- Others (Please specify) (9) ________________________________________________

Q54 Dalam bahagian sebelum ini, anda telah memilih 'Sentiasa', 'Kerap kali‘, 'Kadangkala', atau ’Jarang-jarang/ tidak pernah' untuk tabiat penjagaan diri yang berikut:

 Mengehadkan pengambilan garam, dan kalori dari lemak dan makanan goreng

Jika **sentiasa atau kerap** melakukannya, apakah mendorong anda melibatkan diri dalam tabiat tersebut? ATAU
Jika **kadangkala, jarang-jarang atau tidak pernah** melakukannya, apakah menghalang anda dari sentiasa melibatkan diri dalam tabiat tersebut?
   *Sila pilih maksimum tiga faktor yang paling mempengaruhi tingkah laku anda.*

- Menjaga kesihatan (1)
- kurangkan berat badan (2)
- mencegah pelbagai penyakit (3)
- pilihan makanan yang terhad apabila makan di luar (4)
- makanan sihat lebih mahal (5)
- tidak ada masa untuk menyediakan makanan sihat, makanan segera adalah alternatif yang lebih cepat (6)
- berkongsi makanan dengan keluarga saya memang mencabar (7)
- Saya lebih suka rasa makanan masin/ goreng (8)
- Lain-lain (Sila nyatakan) (9) ________________________________________________

Q54 在前一部分，您为以下的自我保健行为选择了“总是”，“时常”，“有时”或“很少/从来没有”：
   **限制脂肪和油炸食品的盐分和卡路里摄入量**

 若您总是或时常进行，是什么促进您这样做？或若您有时，很少或从来没有进行，是什么阻止您时常这样做？
 *请选择不多于3 最适合您的原因。若以下的原因都不适合请另行写下自己的原因。*

- 维持身体健康 (1)
- 减肥 (2)
- 降低患病的风险 (3)
- 在外饮餐时，有限的饮餐选择 (4)
- 健康食品比较贵 (5)
- 缺乏时间准备健康食品，熟食或快餐是更快的选择 (6)
- 与家人共享一样的饮食增加挑战性 (7)
- 我比较喜欢咸或油炸食品的味道 (8)
- 其他(请明确说明) (9) ________________________________________________

| Page Break |  |
| --- | --- |

| 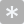 |
| --- |

Q55 In the previous section, you have selected '*Always*', '*Often*', '*Sometimes*', or '*Rarely/never*' for the following self-care behaviour:

 **Eat 5 or more servings of fruits and vegetables daily**

 If you **often or always** do so, what motivates you to engage in the behaviour? OR
 If you **sometimes, rarely or never** do so, what prevents you from always engaging in the behaviour?

 *Please select a maximum of three* factors that influences your behaviour the most.

- improve and maintain health (1)
- prevent various disease (2)
- to lose weight (3)
- I do not like the taste of fruits or vegetables (4)
- limited food choices when eating out (5)
- Others (Please specify) (6) ________________________________________________

Q55 Dalam bahagian sebelum ini, anda telah memilih 'Sentiasa', 'Kerap kali‘, 'Kadangkala', atau ’Jarang-jarang/ tidak pernah' untuk tabiat penjagaan diri yang berikut:
 Makan 5 atau lebih hidangan buah-buahan dan sayur-sayuran setiap hari

Jika **sentiasa atau kerap** melakukannya, apakah mendorong anda melibatkan diri dalam tabiat tersebut? ATAU
Jika **kadangkala, jarang-jarang atau tidak pernah** melakukannya, apakah menghalang anda dari sentiasa melibatkan diri dalam tabiat tersebut?
   *Sila pilih maksimum tiga faktor yang paling mempengaruhi tingkah laku anda.*

- menjaga dan meningkatkan kesihatan (1)
- mencegah pelbagai penyakit (2)
- kurangkan berat badan (3)
- Saya tidak suka rasa buah-buahan atau sayur-sayuran (4)
- pilihan makanan yang terhad apabila makan di luar (5)
- Lain-lain (Sila nyatakan) (6) ________________________________________________

Q55 在前一部分，您为以下的自我保健行为选择了“总是”，“时常”，“有时”或“很少/从来没有”：
   **每日吃5 份或以上的蔬菜水果**

 若您总是或时常进行，是什么促进您这样做？或若您有时，很少或从来没有进行，是什么阻止您时常这样做？
 *请选择不多于3 最适合您的原因。若以下的原因都不适合请另行写下自己的原因。*

- 改善和维持健康 (1)
- 降低患病的风险 (2)
- 减肥 (3)
- 我不喜欢蔬菜水果的味道 (4)
- 在外饮餐时，有限的饮餐选择 (5)
- 其他(请明确说明) (6) ________________________________________________

| Page Break |  |
| --- | --- |

| 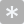 |
| --- |

Q56 In the previous section, you have selected '*Always*', '*Often*', '*Sometimes*', or '*Rarely/never*' for the following self-care behaviour:

 **Practice moderation in drinking alcohol (2 glasses or less for men; 1 glass or less for women?)**

 If you **often or always** do so, what motivates you to engage in the behaviour? OR
 If you **sometimes, rarely or never** do so, what prevents you from always engaging in the behaviour?

 *Please select a maximum of three* factors that influences your behaviour the most.

- religious practices (1)
- I do not like the taste of alcohol (2)
- I know that excessive drinking is bad for health (3)
- festive seasons or events (eg weddings, company dinners) (4)
- it is a stress relief for me (5)
- peer pressure (6)
- Others (Please specify) (7) ________________________________________________

Q56 Dalam bahagian sebelum ini, anda telah memilih 'Sentiasa', 'Kerap kali‘, 'Kadangkala', atau ’Jarang-jarang/ tidak pernah' untuk tabiat penjagaan diri yang berikut:
   **Mengamalkan kesederhanaan dalam pengambilan alkohol setiap hari (2 gelas atau kurang untuk lelaki; 1 gelas atau kurang untuk wanita)**

Jika **sentiasa atau kerap** melakukannya, apakah mendorong anda melibatkan diri dalam tabiat tersebut? ATAU
Jika **kadangkala, jarang-jarang atau tidak pernah** melakukannya, apakah menghalang anda dari sentiasa melibatkan diri dalam tabiat tersebut?
 *Sila pilih maksimum tiga faktor yang paling mempengaruhi tingkah laku anda.*

- amalan agama (1)
- Saya tidak suka rasa alkohol (2)
- Saya tahu bahawa minum yang berlebihan tidak baik untuk kesihatan (3)
- musim perayaan atau acara tertentu (cth majlis perkahwinan, makan malam syarikat) (4)
- ia adalah cara untuk menghilangkan tekanan bagi saya (5)
- tekanan rakan sebaya (6)
- Lain-lain (Sila nyatakan) (7) ________________________________________________

Q56 在前一部分，您为以下的自我保健行为选择了“总是”，“时常”，“有时”或“很少/从来没有”：
   **适当的饮酒（男人不多于2杯，女人不多于1杯）**

 若您总是或时常进行，是什么促进您这样做？或若您有时，很少或从来没有进行，是什么阻止您时常这样做？
 *请选择不多于3 最适合您的原因。若以下的原因都不适合请另行写下自己的原因。*

- 宗教习俗 (1)
- 我不喜欢酒精的味道 (2)
- 我明白酗酒对健康有害 (3)
- 佳节或活动（例：婚礼，公司晚宴） (4)
- 纾解压力 (5)
- 同辈压力 (6)
- 其他(请明确说明) (7) ________________________________________________

| Page Break |  |
| --- | --- |

| 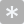 |
| --- |

Q57 In the previous section, you have selected '*Always*', '*Often*', '*Sometimes*', or '*Rarely/never*' for the following self-care behaviour:

 **Check your blood pressure at home or at a clinic (at least once a year)**

 If you **often or always** do so, what motivates you to engage in the behaviour? OR
 If you **sometimes, rarely or never** do so, what prevents you from always engaging in the behaviour?

 *Please select a maximum of three* factors that influences your behaviour the most.

- part of routine checkup when I visit my doctor (1)
- requested by my family (2)
- free blood pressure screenings in my community pharmacy (3)
- I think I am healthy and I do not see a need (4)
- never thought about it (5)
- afraid to know my health status because knowing gives me anxiety (6)
- Others (Please specify) (7) ________________________________________________

Q57 Dalam bahagian sebelum ini, anda telah memilih 'Sentiasa', 'Kerap kali‘, 'Kadangkala', atau ’Jarang-jarang/ tidak pernah' untuk tabiat penjagaan diri yang berikut:
Memeriksa tekanan darah anda di rumah ataupun di klinik (sekurang-kurangnya sekali setahun)
Jika **sentiasa atau kerap** melakukannya, apakah mendorong anda melibatkan diri dalam tabiat tersebut? ATAU
Jika **kadangkala, jarang-jarang atau tidak pernah** melakukannya, apakah menghalang anda dari sentiasa melibatkan diri dalam tabiat tersebut? *Sila pilih maksimum tiga faktor yang paling mempengaruhi tingkah laku anda.*

- sebahagian daripada pemeriksaan rutin apabila saya berjumpa dengan doktor saya (1)
- diminta oleh keluarga saya (2)
- pemeriksaan tekanan darah percuma (cth. di farmasi komuniti) (3)
- Saya fikir saya sihat dan saya tidak memerlukannya (4)
- tidak pernah memikirkannya (5)
- takut mengetahui status kesihatan kerana mengetahuinya akan membimbangkan saya (6)
- Lain-lain (Sila nyatakan) (7) ________________________________________________

Q57 在前一部分，您为以下的自我保健行为选择了“总是”，“时常”，“有时”或“很少/从来没有”：
   **在家或诊所检查自己的血压（一年至少1次）**

 若您总是或时常进行，是什么促进您这样做？或若您有时，很少或从来没有进行，是什么阻止您时常这样做？
 *请选择不多于3 最适合您的原因。若以下的原因都不适合请另行写下自己的原因。*

- 看医生进行例行检查的一部分 (1)
- 被家人要求 (2)
- 我的社区药房提供免费血压筛查 (3)
- 我认为我很健康，因此觉得没必要 (4)
- 没想过 (5)
- 害怕知道我的健康状况，因为知道会使我焦虑 (6)
- 其他(请明确说明) (7) ________________________________________________

| Page Break |  |
| --- | --- |

End of Block: Factors

Start of Block: Complaints and contacts

Q52
**Thank you for participating in this research**!
 
In case you have a complaint about the study:
 You can approach the researcher at khyy6ptp@nottingham.edu.my, 

 If this achieves no satisfactory outcome, you can contact the secretary of the Ethics Committee, of the Faculty of Science and Engineering, UNM at vanitha.singaram@nottingham.edu.my.  
*Please click the "next" button to end and submit the survey.*

Q52
**Terima kasih atas bantuan dengan penyelidikan ini**

 Sekiranya anda mempunyai aduan mengenai kajian ini, anda boleh menghubungi penyiasat di khyy6ptp@nottingham.edu.my


Sekiranya ini tidak mencapai hasil yang memuaskan, anda boleh menghubungi setiausaha Jawatankuasa Etika, Fakulti Sains dan Kejuruteraan, UNM di vanitha.singaram@nottingham.edu.my.
 
*Sila klik butang "seterusnya" untuk menamatkan dan menyerahkan soal selidik.*

Q52
 **感谢您参与这项研究！**    
 若您对于这份问卷有任何的不满：
  您可以联络我们的研究员 khyy6ptp@nottingham.edu.my,   若无法取得满意的结果，可以通过vanitha.singaram@nottingham.edu.my联系UNM科学与工程学院道德委员会秘书

 *请单击右下角的按钮以结束及提交问卷。*

End of Block: Complaints and contacts
